# Development and Validation of a Centrosome Amplification-Related Prognostic Model in Pancreatic Cancer: Multi-Omics Guided Risk Stratification and Tumor Microenvironment

**DOI:** 10.3390/cancers17182983

**Published:** 2025-09-12

**Authors:** Yuan Sun, Tao Hu, Yan Li, Ming Li

**Affiliations:** Department of Hepatobiliary Surgery, The First Affiliated Hospital of Chongqing Medical University, Chongqing 400016, China; 2023120494@stu.cqmu.edu.cn (Y.S.); 2023150447@stu.cqmu.edu.cn (T.H.); 2023120470@stu.cqmu.edu.cn (Y.L.)

**Keywords:** pancreatic cancer, centrosome amplification, prognosis diagnosis, tumor microenvironment, drug sensitivity

## Abstract

**Background:** Centrosome amplification, a hallmark of cell cycle dysregulation, drives carcinogenesis through aneuploidy induction and invasive phenotype acquisition. In pancreatic adenocarcinoma—a malignancy characterized by profound genomic instability—the molecular circuitry of centrosome amplification remains enigmatic. Critical gaps persist in understanding its spatiotemporal dynamics in tumor microenvironment remodeling and therapy resistance. **Methods:** This study integrated centrosome amplification-related genes from TCGA and Genecards, established a prognostic risk model through univariate Cox regression–LASSO penalized Cox regression–multivariate Cox regression analyses, and validated it using GEO datasets. Single-cell sequencing analyses dissected transcriptional heterogeneity and intercellular communication networks, while spatially resolved transcriptomics unveiled spatiotemporal expression patterns and molecular regulatory mechanisms of core genes. With further experimental validation via PCR analysis of patient-derived tissue samples confirming key gene expression patterns. **Results:** This study identified 23 centrosome amplification-related prognostic genes in pancreatic adenocarcinoma, establishing *IFI27*, *KIF20A*, *KLK10*, *SPINK7*, and *TOP2A* as highly specific diagnostic and prognostic biomarkers. The constructed signature was established as an independent prognostic indicator correlating with aggressive clinicopathological characteristics and chemoresistance. Mechanistically linked to enhanced DNA repair capacity and accelerated cell cycle progression, also synergizes with KRAS mutational profiles. Tumor microenvironment analysis revealed significant associations with immunosuppressive. Single-cell resolution demonstrated cellular specificity of IFI27/KLK10 in ductal epithelial cells and fibroblasts, with intercellular communication networks exhibiting multidimensional regulatory features. Spatially resolved transcriptomics delineated tumor-region-specific expression patterns of core genes. While PCR validation on matched tumor/normal tissues confirmed significant differential expression of *IFI27*, *KIF20A*, *KLK10*, and *TOP2A*. **Conclusions:** This study deciphers the multidimensional clinic–molecular network orchestrated by centrosome amplification in PDAC, revealing its dual-pathogenic mechanism in fueling tumor aggressiveness through coordinated induction of genomic instability and immunosuppressive microenvironment reprogramming. These findings establish a translational framework for developing centrosome dynamics-based prognostic stratification and molecularly targeted therapeutic strategies.

## Simple Summary

This study deciphers the critical role of centrosome amplification in driving pancreatic adenocarcinoma (PAAD) progression and therapy resistance. By integrating multi-omics analyses (TCGA, GEO, single-cell/spatial transcriptomics) and experimental validation, we identified 23 centrosome amplification-related prognostic genes, with *IFI27*, *KIF20A*, *KLK10*, *SPINK7*, and *TOP2A* serving as highly specific diagnostic and prognostic biomarkers. The established gene signature correlates with aggressive tumor behavior, chemoresistance, and immunosuppressive microenvironment remodeling. Mechanistically, these genes synergize with KRAS mutations to accelerate cell cycle progression and enhance DNA repair. Single-cell and spatial analyses revealed cellular specificity (e.g., IFI27/KLK10 in ductal cells/fibroblasts) and tumor-region-specific expression patterns. These findings provide a translational framework for centrosome-targeted prognostic stratification and therapies in PAAD.

## 1. Introduction

Pancreatic cancer is among the most malignant tumors of the digestive system, and surgical resection remains the preferred therapeutic strategy for pancreatic adenocarcinoma (PAAD). Due to the extremely poor prognosis of this invasive disease, compounded by the fact that most patients are diagnosed at an advanced stage, only 15–20% of patients are eligible for surgical intervention at diagnosis, leading to one-year and five-year survival rates of 24% and 9%, respectively [1]. In recent years, the combination of first-line chemotherapy regimens such as FOLFIRINOX with agents including nivolumab (a PD-1 inhibitor) and nimotuzumab (an EGFR inhibitor) has modestly improved the survival rate of patients with PAAD. However, the overall therapeutic efficacy remains unsatisfactory [2,3]. Consequently, there is an urgent need to develop new therapeutic targets and establish prognostic models capable of evaluating patient outcomes to guide clinical decision-making.

Centrosome amplification—an aberrant increase in the number of centrosomes within a cell—has been recognized as a hallmark of cancer in recent years [4]. However, the specific factors that induce centrosome amplification and how they influence tumor development remain to be fully elucidated. Existing research indicates that alterations in the proteins involved in centrosome replication can trigger abnormal centrosome amplification. For instance, dysregulation of PLK4 disrupts centriole replication and causes abnormal numbers of centrosomes; Mittal K et al. identified a hypoxia-inducible factor 1α (HIF1α)/PLK4 axis that drives centrosome amplification in cancer cells, promoting cell migration and invasion [5]. Additionally, one study demonstrated that knocking down cell division cycle protein6 (Cdc6) promotes apoptotic cell death, accompanied by the activation of calpain-1 and caspase-9; these processes play crucial roles in the proliferation, cell cycle progression, and death of pancreatic cancer cells [6]. Nevertheless, most previous work has focused on the functions of individual genes rather than on a global transcriptomic analysis of centrosome amplification-related genes (CARGs). Therefore, a systematic investigation of CARGs in PAAD is warranted to gain novel insights into the underlying mechanisms of tumorigenesis and to identify new targets for cancer prevention and therapy.

In this study, we performed a comprehensive analysis of CARGs in 177 PAAD patients from The Cancer Genome Atlas (TCGA) dataset. We initially retrieved 724 Centrosome amplification-related genes from the GeneCards database. Of these, 23 showed elevated expression in PAAD tissues. Subsequently, using univariate Cox, LASSO-penalized Cox regression and multivariate Cox, we identified a five-gene signature (*IFI27*, *KIF20A*, *KLK10*, *SPINK7*, and *TOP2A*) from these 23 CARGs. We next investigated the associations between this centrosome amplification-related prognostic signature and immune infiltration, pathway enrichment, genetic mutations, chemosensitivity in pancreatic cancer, single-cell RNA sequencing analysis and spatial transcriptomic analysis. Finally, qPCR validation was performed using surgical specimens of pancreatic cancer.

## 2. Materials and Methods

### 2.1. Data Sources

We collected clinical and pathological information (including age, sex, tumor grade, tumor stage, tumor type, tumor location, TNM stage, survival time, and vital status) for 177 pancreatic cancer (PAAD) tissue samples retrieved from The Cancer Genome Atlas (TCGA) database (originally 178 samples, with one excluded due to missing survival data). We also obtained the corresponding transcriptome sequencing data (seq-TPM, seq-count) for these 177 PAAD samples (22 March 2025). In addition, transcriptome sequencing data (seq-TPM, seq-count) for 180 normal pancreatic tissues were downloaded from the GTEx database, and all transcriptomic data underwent normalization. Furthermore, GSE183795 and GSE62452 from the GEO database were selected as validation cohorts, and GSE155698 (comprising single-cell RNA sequencing [scRNA-seq] data from 10 PAAD tumor samples) was used for single-cell analysis. GSE274557 (spatial transcriptomics data) was used for spatial transcriptomic analysis.

### 2.2. Methods

#### 2.2.1. Differential Gene Expression Analysis Between PAAD and Non-Tumor Tissues and Identification of Centrosome Amplification-Related Differential Genes

To identify transcriptional differences between PAAD and non-tumor tissues, we downloaded 177 PAAD tumor samples from The Cancer Genome Atlas (TCGA) database (https://www.cancer.gov/ccg/research/genome-sequencing/tcga, accessed on 22 March 2025) and 180 normal pancreatic tissue samples from the Genotype-Tissue Expression (GTEx) database (https://gtexportal.org/home/, accessed on 22 March 2025). Using the DESeq2 package in R, we screened for differentially expressed genes (DEGs) between tumor and non-tumor samples, applying the thresholds |fold change| > 1.5 and adjusted *p* < 0.05. The ggplot package (version 3.5.0) was used to visualize these DEGs. From GeneCards (https://www.genecards.org/, accessed on 22 March 2025), we retrieved 739 centrosome amplification-related genes (relevance score > 1.2). We then used the VennDiagram package (version 1.7.3) in R to identify and visualize overlapping genes between the PAAD DEGs and the centrosome amplification-related gene set. Ultimately, 23 genes that were both upregulated in PAAD and associated with centrosome amplification were selected for subsequent analyses.

#### 2.2.2. Construction and Validation of a Prognostic Signature Based on Centrosome Amplification-Related Genes

To construct a prognostic model based on the aforementioned 23 CARGs, we first employed a univariate Cox proportional hazards model to identify prognostic-related genes (PRGs) among the CARGs, with a *p*-value < 0.05 considered statistically significant. We then performed least absolute shrinkage and selection operator (LASSO) Cox regression using the “glmnet” package in R (alpha = 1, where alpha = 1 specifies the exclusive use of L1 penalty: λ × ∑|β|) to minimize overfitting risks and identify the most significant survival-associated CARGs in PAAD; specifically, we employed 10-fold cross-validation via cv.glmnet (family = “cox”, nfolds = 10, nlambda = 100) to determine the optimal regularization parameter λ, which automatically shrinks coefficients of non-contributory variables to zero through L1 penalization while selecting the λ value that maximizes generalizability based on validation across independent subsets. Subsequently, a multivariate Cox proportional hazards analysis was implemented with bidirectional stepwise regression (step function, direction = “both”) to refine the model, where only variables meeting statistical significance (*p* < 0.05) were retained to establish the finalized coefficients (β) for each gene incorporated into the prognostic signature, thereby instituting a dual-safeguard mechanism against model overfitting. The following formula was used to calculate each patient’s risk score in the TCGA dataset:

Risk Score = βmRNA 1 × Expression of mRNA 1 + βmRNA 2 × Expression of mRNA 2 + … + βmRNA *n* × Expression of mRNA *n*

Using this risk prediction model, we calculated the risk score for each patient. All patients in the TCGA-PAAD dataset were then classified into low-risk or high-risk groups based on the median risk score. Next, we applied the “survival” and “survminer” packages in R to generate Kaplan–Meier survival curves and perform log-rank tests, evaluating the survival differences between the two groups. Additionally, receiver operating characteristic (ROC) curves were generated, and the area under the ROC curve (AUC) was determined using the “timeROC” package to assess the sensitivity and specificity of the prognostic model in predicting 2-, 3-, and 5-year survival rates. Furthermore, we downloaded another independent PAAD dataset from GSE78229, GSE62452, and GSE183795 to validate the robustness and effectiveness of the CARG-based prognostic model.

Next, both univariate and multivariate Cox proportional hazards models were employed to assess whether the CARG-based prognostic model demonstrates robust predictive power independent of other clinicopathological characteristics, including age, sex, grade, T stage, M stage, N stage, history of previous malignancy, tumor status at follow-up, tumor location, DCC histological subtype, history of radiotherapy, residual tumor, and history of chronic pancreatitis. Subsequently, a prognostic nomogram was constructed by integrating variables identified in the univariate model. A calibration curve was then generated to evaluate the concordance between the nomogram-predicted survival outcomes and the actual observations.

#### 2.2.3. Identification of Differentially Expressed Genes Between High- and Low-Risk Groups and Functional Enrichment Analysis

To identify the differentially expressed genes (DEGs) between high- and low-risk PAAD patient groups defined by the predictive model, we used DESeq2 with ∣fold-change∣ > 1 and adjusted *p* < 0.05 as cutoffs. These DEGs (referred to as DEGs 2) were then subjected to Gene Ontology (GO) and Kyoto Encyclopedia of Genes and Genomes (KEGG) enrichment analyses using the ClusterProfiler package to further explore their biological functions. The GO analysis covered cellular components (CC), biological processes (BP), and molecular functions (MF), and the results were recorded accordingly.

To estimate the biological functions and signaling pathways associated with the centrosome amplification-related gene signature, we conducted Gene Set Variation Analysis (GSVA). Specifically, we used the GSVA package to score each patient’s molecular features in the TCGA dataset, employing the gene set h.all.v7.0.symbols.gmt. Significant pathways (adjusted *p* < 0.05) between the high- and low-risk groups were assessed using the DESeq2 package.

To further analyze the functional enrichment of the DEGs in various biological pathways, we performed Gene Set Enrichment Analysis (GSEA). First, we ranked the DEGs based on their log2FoldChange values. Then, we carried out GSEA to identify significantly enriched pathways.

#### 2.2.4. Tumor Microenvironment and Immune Infiltration Analysis

We employed the CIBERSORT algorithm to deconvolute the immune microenvironment of tumor samples, estimating the infiltration levels of 22 immune cell types. We compared immune cell infiltration levels between high- and low-risk groups using box plots generated by the ggpubr(version 0.6.1) and reshape2 packages(version 0.8.10), with statistical significance evaluated via the Wilcoxon rank-sum test. Spearman’s correlation analysis was applied to assess the relationships between the risk score and the 22 immune cell types, as well as between the expression of the five signature genes and these cell types. Next, we performed single-sample Gene Set Enrichment Analysis (ssGSEA) to derive enrichment scores for 28 immune cell types and their associated immune functions, then compared immune checkpoint expression between the two risk groups. We also utilized the ESTIMATE algorithm to quantify tumor purity, immune infiltration, and stromal infiltration, and examined correlations between immune/stromal scores and the risk score using scatter plots and linear regression. To estimate immunotherapy responses, we applied the TIDE (Tumor Immune Dysfunction and Exclusion) algorithm and visualized the results. We further analyzed correlations between key genes and immune cells. In addition, we leveraged the TCIA database to examine immune scores under various immune checkpoint inhibitor regimens (e.g., CTLA4 and PD-1), comparing high- versus low-risk groups via box plots and the Wilcoxon rank-sum test.

#### 2.2.5. Mutation Analysis

Using the “maftools(version 2.20.0),” “remodeling2 (version 1.0.0),” and “ggpubr” packages(version 0.6.0) in R, we evaluated and compared the mutation burden and mutation profiles of the high- and low-risk groups, and generated waterfall plots to visualize the main mutated genes in each group. We then integrated the risk scores with tumor mutation burden (TMB) data, compared TMB levels between the two risk groups, and visualized the results in box plots (using ggpubr(version 0.6.0)) with statistical significance testing. Based on the median TMB, samples were further divided into high- and low-TMB groups. A chi-square test was conducted to assess the distribution of high and low TMB across the two risk groups, with proportion bar plots for visualization.

#### 2.2.6. Drug Sensitivity Evaluation

The oncoPredict R package (version 0.2.3) predicts clinical drug responses based solely on baseline tumor gene expression data. This is accomplished via statistical models trained on cell line gene expression and drug sensitivity data from the Cancer Genome Project (CGP). Using oncoPredict in R, we estimated half-maximal inhibitory concentrations (IC50) for 198 chemotherapy and targeted drugs for each patient in the TCGA-PAAD dataset (n = 177). Patients were stratified into a high-risk group (n = 88) and a low-risk group (n = 89), and IC50 values were determined accordingly.

#### 2.2.7. Single-Cell RNA Sequencing Analysis

Single-cell RNA-seq (scRNA-seq) data were obtained from the Gene Expression Omnibus (GEO) database under accession GSE155698 (https://www.ncbi.nlm.nih.gov/geo/query/acc.cgi?acc=GSE155698, on 22 March 2025), comprising 17 PAAD tumor samples. Data processing was carried out using the “Seurat v4.3.0” R package. Cells expressing fewer than 200 or more than 5000 genes, or those with >5% mitochondrial genes, were excluded. We applied “LogNormalize” (scale factor = 10,000), identified 2000 highly variable genes, and scaled these genes. Principal component analysis (PCA) was used for dimension reduction, followed by batch effect correction via the Harmony algorithm (v1.0), which designated sample origin (orig.ident) as the grouping variable (group.by.vars = “orig.ident”). This entailed nonlinearly mapping heterogeneous PCA embeddings onto a unified reference space to eliminate technical biases. Clusters were identified using harmony-corrected embeddings (top 20 dimensions), and visualized via t-SNE. Cell clusters were annotated based on known marker genes. To examine prognostically relevant genes, we used “FeaturePlot” to visualize their expression on t-SNE plots. Gene sets were defined, module scores calculated using AddModuleScore, and integrated into single-cell data. The “FeaturePlot” and “VlnPlot” functions displayed score distributions across clusters.

#### 2.2.8. Cell–Cell Communication

Cell–cell communication analysis was performed using the CellChat R package (v1.6.1) to decode signaling networks based on known ligand–receptor interactions and cofactors. Phenotype-driven cell subgrouping where a 5-gene signature quantified module scores within ductal epithelial cells, stratifying them into high/low-score subgroups via median thresholds; Systematic ligand–receptor (L-R) inference involving identification of overexpressed ligands/receptors across all subgroups (including score-stratified populations), computation of communication probabilities using CellChatDB.human-validated pairs, and statistical filtering of interactions through permutation testing (*p* < 0.05); Network characterization wherein statistically significant L-R-mediated interactions were visualized via customized heatmaps and topological diagrams, complemented by centrality analyses to identify dominant signaling roles. Bubble charts and centrality plots further elucidated interaction patterns, specifically enabling comparison of phenotype-dependent communication states within ductal epithelial compartments.

#### 2.2.9. Spatial Transcriptomics Analysis

Spatial transcriptomic (ST) data (GSE274557) were processed through an integrated computational pipeline: initial quality control involved spot-level filtering based on nCount_Spatial and nFeature_Spatial thresholds to exclude low-quality regions, followed by SCTransform normalization (Seurat v5) and PCA-based feature selection (dims = 1:15). Unsupervised clustering (FindClusters, resolution = 0.8) and UMAP visualization revealed spatial domains, while RCTD-based integration with GSE155698 scRNA-seq data enabled cell-type mapping and core gene localization. Ductal epithelial trajectories were reconstructed via DDRTree pseudotime analysis (Monocle3), and SpaCET with TCGA PAAD reference data identified malignant cell distributions. Tumor-stroma interfaces were delineated through topological feature extraction, with multi-core parallelization (8 threads) accelerating subclone refinement. Spatial colocalization networks were statistically validated by permutation testing (FDR < 0.05) and visualized using Cytoscape (https://cytoscape.org/), revealing *IFI27/KLK10* expression gradients across tumor niches.

#### 2.2.10. Quantitative Real-Time PCR Analysis and HPA Immunohistochemistry Validation

Total RNA was extracted using the Trizol method, and its integrity was confirmed by agarose gel electrophoresis. The first-strand cDNA was synthesized using the Advantage^®^ RT-for-PCR Kit (Takara, Tokyo, Japan). Quantitative real-time PCR was performed on an Applied Biosystems QuantStudio 5 system with a 20 μL reaction mixture containing AceQ^®^ SYBR^®^ Green Master Mix (Vazyme, Nanjing, China), 0.4 μM gene-specific primers, and diluted cDNA templates. The thermal cycling protocol included an initial pre-denaturation step at 95 °C for 5 min, followed by 40 cycles of 95 °C for 15 s and 60 °C for 30 s. Data analysis was conducted using the 2^−ΔΔCt^ method: ΔCt values were calculated as Ct (target gene) − Ct (GAPDH) to normalize inter-sample variations, ΔΔCt was defined as ΔCt (tumor tissue) − ΔCt (paired adjacent normal tissues), and the relative fold change in gene expression was determined by 2^−ΔΔCt^. Additionally, immunohistochemistry (IHC) data were downloaded from the Human Protein Atlas (HPA, http://www.proteinatlas.org) to evaluate the high and low expression patterns of 5 genes in pancreatic cancer tissues.

### 2.3. Statistical Analysis

All statistical analyses were performed using R (versions 4.1.3 and 4.3.2) along with relevant R packages. Kaplan–Meier survival curves were generated, and log-rank tests were used to compare survival differences across risk groups. Univariate and multivariate Cox regression models were constructed to evaluate the prognostic value of clinicopathological variables and risk scores. Chi-square or Fisher’s exact tests were employed to examine relationships between risk scores and clinical characteristics. Group comparisons were conducted using the Mann–Whitney U test (Wilcoxon rank-sum test). A two-sided *p*-value < 0.05 was considered statistically significant (* *p* < 0.05; ** *p* < 0.01; *** *p* < 0.001; **** *p* < 0.0001).

## 3. Results

### 3.1. Identification of Differentially Expressed Genes in Pancreatic Cancer

**Figure 1:**
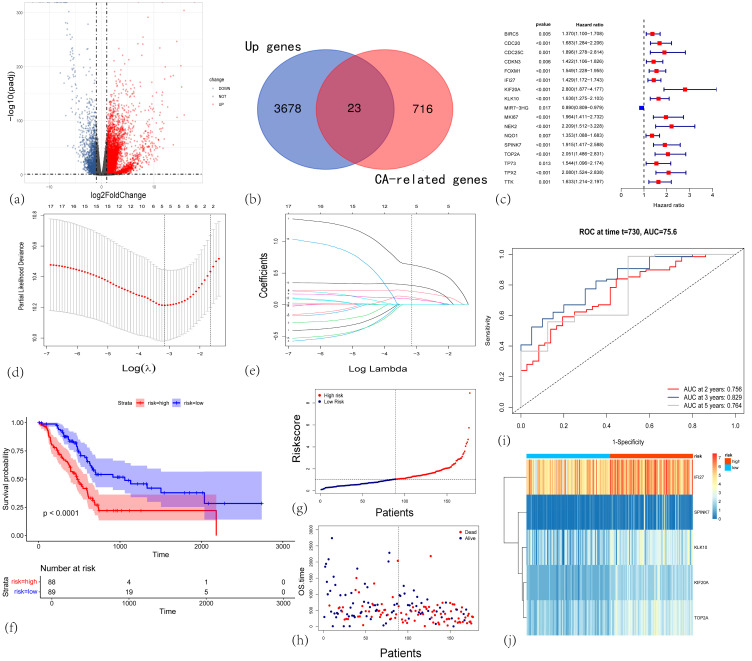
(**a**) Volcano plot illustrating differentially expressed genes (DEGs) between PAAD tumor tissues and normal pancreatic tissues. (**b**) Venn diagram showing the intersection between upregulated DEGs (blue circle) and centrosome amplification-related genes (red circle). (**c**) Forest plot from univariate Cox proportional hazards analysis of the centrosome amplification-related genes in PAAD. (**d**) LASSO regression model selection using 10-fold cross-validation. (**e**) Coefficient profiles of the survival-associated genes as a function of log (λ). (**f**) Kaplan–Meier survival curves for the high-risk (red, n = 88) and low-risk (blue, n = 89) groups in the TCGA-PAAD cohort. (**g**) Risk score distribution plot. Each dot represents an individual patient. (**h**) Scatter plot showing each patient’s survival status (alive or dead) and OS time. (**i**) Time-dependent ROC curves for 2-, 3-, and 5-year OS prediction. (**j**) Heatmap illustrating the expression patterns of the five signature genes in high-risk (red) and low-risk (blue) patients.

To uncover key genes involved in the development and progression of PAAD, we first investigated DEGs between 177 PAAD tumor tissues from the TCGA database and 180 normal pancreatic tissues from the GTEx database. As shown in Figure 1a, using the “DESeq2” package (cutoffs: log2FC > 1.5, q < 0.05), we identified 3701 upregulated genes and 924 downregulated genes in pancreatic cancer tissues, yielding a total of 4625 DEGs.

### 3.2. Construction and Validation of a Centrosome Amplification-Related Prognostic Gene Model in PAAD

To identify key centrosome amplification-related genes (CARGs) involved in PAAD progression, we collected a set of CARGs from the GeneCards database. We then generated a Venn diagram to overlap the 739 centrosome replication-associated genes with the 3701 upregulated DEGs, ultimately identifying 23 CARGs (Figure 1b). Principal component analysis (PCA) further demonstrated that these 23 CARGs effectively distinguish non-tumor from tumor samples (Figure S1).

Next, we performed Gene Ontology (GO) enrichment analysis to gain insights into the molecular mechanisms by which these differentially expressed CARGs might contribute to PAAD development. Results showed that these CARGs are enriched in condensed chromosomes, centromeres, microtubules, and the spindle, as well as pathways involving the cell cycle and the Hippo signaling pathway (Figure S2a–d), including processes such as mitotic nuclear division, chromosome segregation, mitotic cell cycle phase transition, and microtubule cytoskeleton organization during mitosis. This suggests a critical role for centrosome amplification in PAAD through cell cycle regulation.

Subsequently, we applied univariate Cox regression to these 23 CARGs and identified 17 prognostic genes (Figure 1c). These genes were further subjected to LASSO-penalized Cox regression (Figure 1d,e) and multivariate Cox regression, yielding 5 final genes—*IFI27*, *KIF20A*, *KLK10*, *SPINK7*, and *TOP2A* (Figure S3)—all of which exhibited higher expression levels in PAAD tissues compared to normal pancreatic tissues. To develop a prognostic risk score, the mRNA expression of each gene was multiplied by its corresponding multivariate Cox coefficient and summed to generate an individual risk score:

Risk score = (0.085 × *IFI27* expression) + (0.631 × *KIF20A* expression) + (0.211 × *KLK10* expression) + (0.305 × *SPINK7* expression) + (0.114 × *TOP2A* expression).

Using the median risk score (2.8052) as the cutoff, patients in the TCGA-PAAD cohort (n = 177) were divided into low-risk (risk score < 2.8052, n = 89) and high-risk (risk score ≥ 2.8052, n = 88) groups. A heatmap revealed higher expression levels of *IFI27*, *KIF20A*, *KLK10*, *SPINK7*, and *TOP2A* in the high-risk group (Figure 1j). Risk score distribution plot and scatter plots indicated that higher risk scores were associated with poorer overall survival (OS) (Figure 1g,h). Consistently, Kaplan–Meier curves and log-rank tests showed significantly worse OS for patients in the high-risk group compared to those in the low-risk group (*p* < 0.0001; Figure 1f). Time-dependent receiver operating characteristic (ROC) curves confirmed that this 5-gene signature had strong prognostic performance, with areas under the ROC curve (AUCs) of 0.752, 0.826, and 0.764 at 2, 3, and 5 years, respectively (Figure 1i).

**Figure 2:**
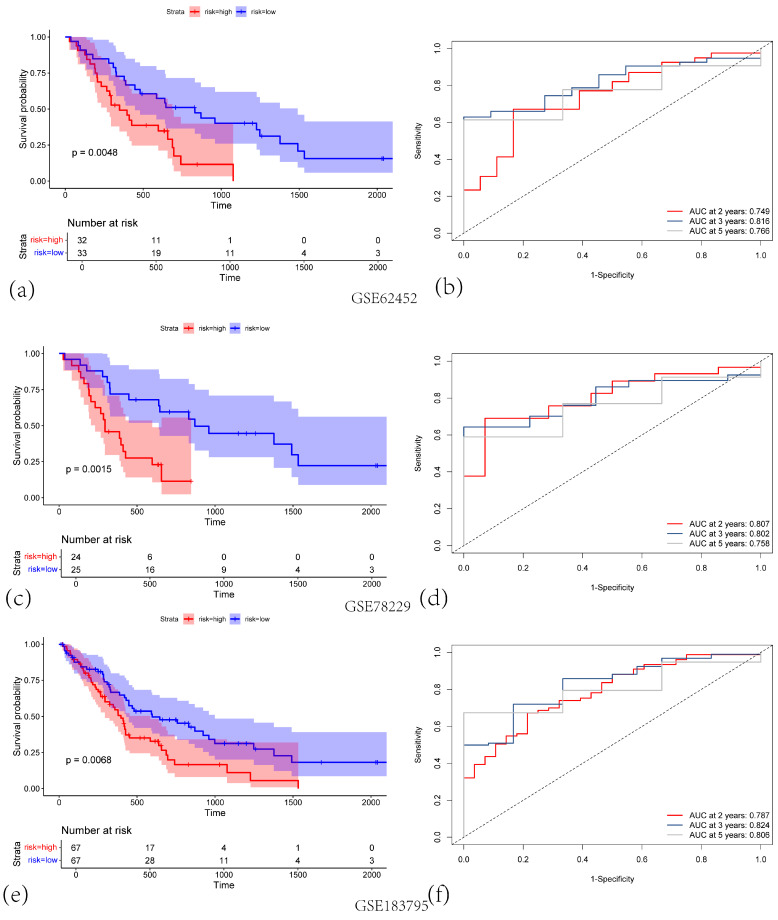
(**a**) KM survival curves for the high-risk (red) and low-risk (blue) groups in GSE62452 validation cohort. (**b**) Corresponding ROC curves for predicting 2-, 3-, and 5-year overall survival (OS), with the respective AUC values indicated in GSE62452 validation cohort. (**c**) KM survival curves for high-risk versus low-risk groups in GSE78229 validation cohort. (**d**) Time-dependent ROC curves at 2, 3, and 5 years, illustrating the predictive performance of the signature in GSE78229 validation cohort. (**e**) KM survival curves showing significant differences in OS between high-risk and low-risk groups in GSE183795 validation cohort. (**f**) ROC curves demonstrating the model’s performance at 2, 3, and 5 years in GSE183795 validation cohort.

We further validated the 5-gene signature in an independent dataset, GSE183795 (n = 134), by stratifying patients according to the median risk score derived from the TCGA cohort. Consistent with the TCGA results, high-risk patients had higher mortality rates and poorer survival outcomes than low-risk patients (*p* < 0.001; Figure 2e). The AUCs for predicting 2-, 3-, and 5-year OS in this validation cohort were 0.787, 0.818, and 0.817, respectively (Figure 2f). Similar findings were obtained in other independent datasets (GSE78299 and GSE62452), as illustrated in Figure 2a–d. Collectively, these results underscore the robust prognostic utility of the 5-gene centrosome amplification-related signature in PAAD.

### 3.3. The Centrosome Amplification-Related Prognostic Signature Is an Independent Prognostic Factor in PAAD

We next evaluated whether the centrosome amplification-related risk score could serve as an independent prognostic factor in PAAD. A univariate Cox regression analysis was performed, incorporating the risk score as well as multiple clinicopathological features—age, sex, tumor grade, T stage, M stage, N stage, history of previous malignancy, tumor status at follow-up, tumor location, DCC histological subtype, history of radiotherapy, residual tumor, and chronic pancreatitis. Univariate Cox results indicated that age (HR = 1.0279, 95% CI: 1.0071–1.0492), N stage (HR = 2.0961, 95% CI: 1.2486–3.5188), histological grade (HR = 2.3502, 95% CI: 1.443–2.287), tumor status at follow-up (HR = 2.4912, 95% CI: 1.0770–5.7623), history of radiotherapy (HR = 0.4525, 95% CI: 0.2566–0.7980), DCC subtype (HR = 1.7700, 95% CI: 1.0773–2.9080), and residual tumor (HR = 1.7703, 95% CI: 1.0971–2.8568) were predictive of clinical outcomes in the TCGA cohort (Figure S4a). In the multivariate Cox regression, the centrosome amplification-related risk score emerged as a significant factor for prognosis, independent of other clinical variables (Figure S4b).

**Figure 3:**
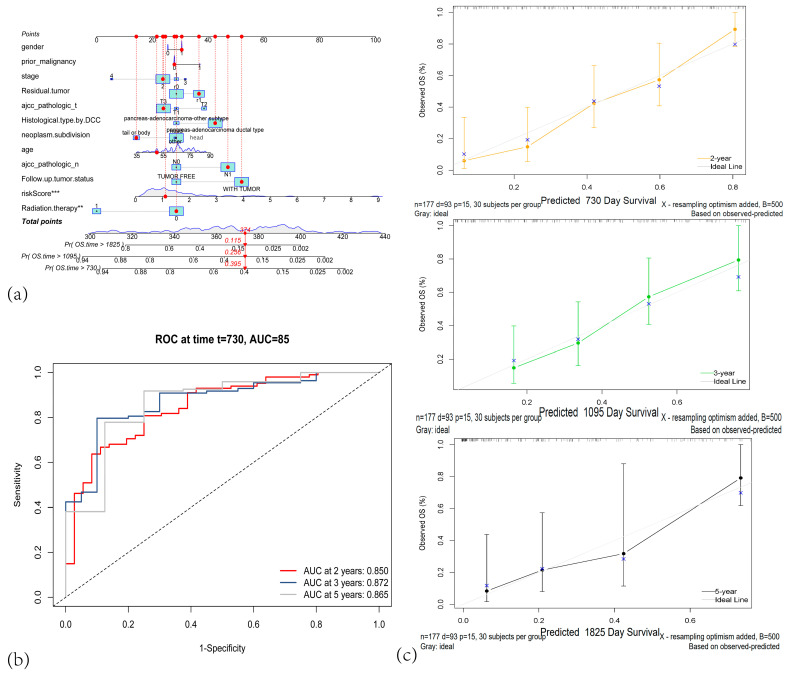
(**a**) Nomogram for predicting 2-year, 3-year, and 5-year overall survival (OS) in PAAD patients. (**b**)Time-dependent ROC curves of the nomogram for predicting 2-year, 3-year, and 5-year survival. (**c**) Calibration plots for predicted survival at 2 years (orange), 3 years (green), and 5 years (black). (** *p* < 0.01; *** *p* < 0.001).

We subsequently combined these 7 variables with the risk score in a multivariate Cox regression and constructed a stepwise prognostic Cox model to predict 2-, 3-, and 5-year OS, culminating in a nomogram (Figure 3a). The nomogram parameters included the risk score, age, N stage, histological grade, tumor status at follow-up, history of radiotherapy, DCC subtype, and residual tumor. The AUCs for predicting 2-, 3-, and 5-year OS based on the nomogram were 0.850, 0.872, and 0.865, respectively (Figure 3b). Calibration curves closely aligned with the 45° diagonal, indicating a strong concordance between nomogram-predicted survival and observed survival in the TCGA-PAAD cohort (Figure 3c).

**Figure 4:**
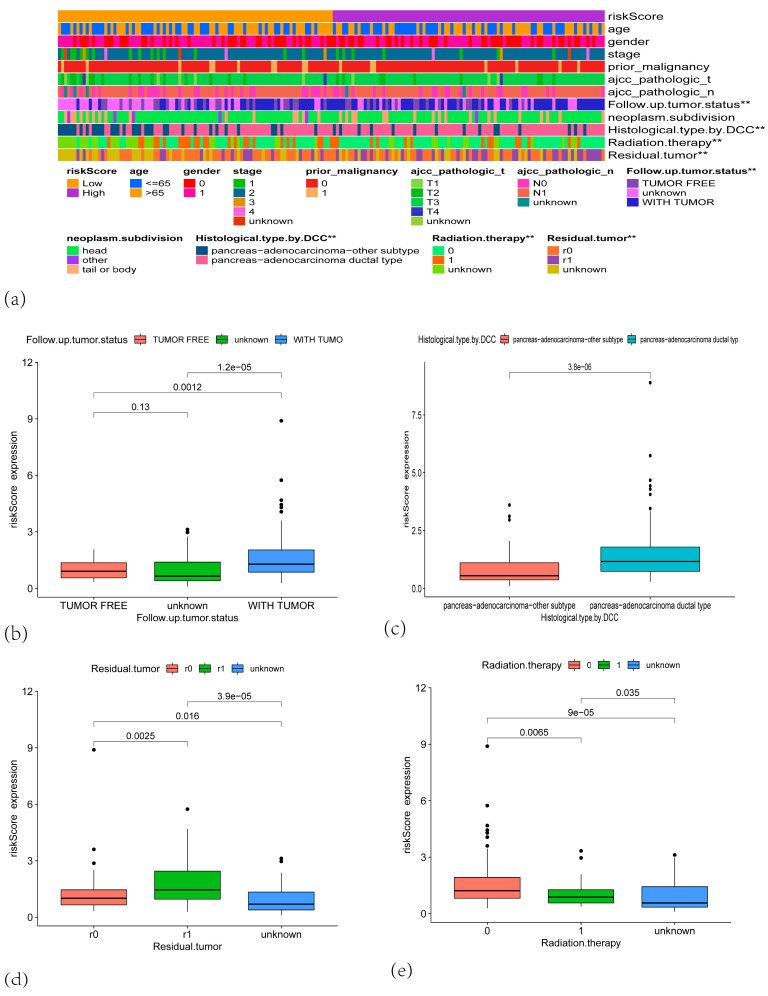
(**a**) A heatmap showing the distribution of clinical features and risk scores across high-risk and low-risk groups. (**b**) Boxplot showing the risk score expression distribution based on the follow-up tumor status. (**c**) Boxplot comparing the risk score expression based on histological type by DCC. (**d**) Boxplot of risk score expression based on residual tumor status. (**e**) Boxplot of risk score expression based on radiation therapy status. ** *p* < 0.01).

Additionally, clinicopathological relevance analyses demonstrated that the risk score was significantly associated with tumor status at follow-up, history of radiotherapy, presence of residual tumor, and DCC histological subtype (Figure 4a–e). The clinicopathological relevance analysis of the 5-gene signature with tumor staging revealed distinct associations: *IFI27* demonstrated positive associations with Stage I–II tumors, lymph node metastasis, and T1/T3 stages; *KLK10* exhibited positive correlations with Stage I–II tumors and T2/T3 stages; *KIF20A* showed significant linkages to Stage I–II tumors and T2/T3 stages; *SPINK7* exclusively correlated with T1, T2, and T4 stages; and *TOP2A* was uniquely associated with T2 and T3 stages (Figure S5).

### 3.4. Correlation Analysis of the CARG Signature with Immune Infiltration and the Tumor Microenvironment

**Figure 5:**
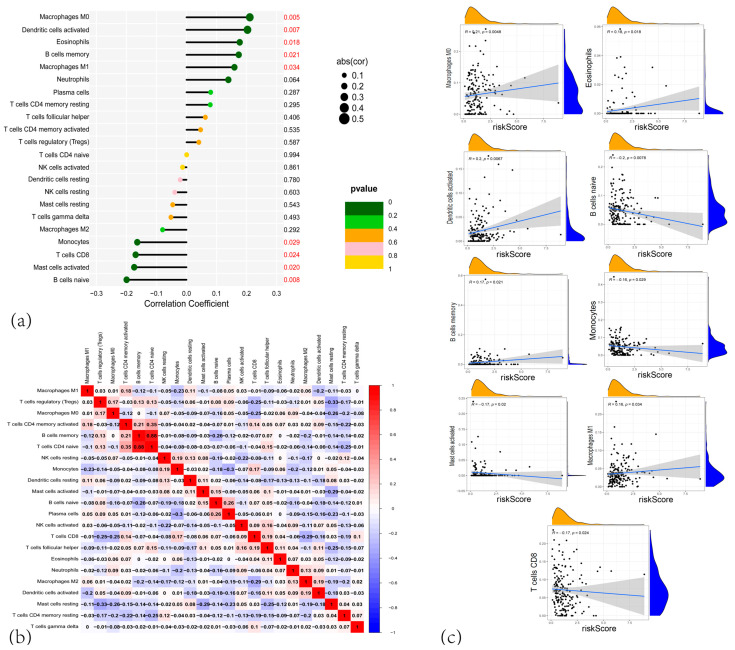
(**a**) Forest plot of the correlation between the proportion of different immune cells and risk scores. (**b**) Heatmap showing the correlation matrix between the different immune cell fractions. (**c**) Scatter plots showing the relationship between risk scores and specific immune cell types.

To explore the relationship between the CARG signature, immune infiltration, and the tumor microenvironment, we performed immune deconvolution analysis on tumor samples using the CIBERSORT algorithm and compared the infiltration levels of immune cells between the high-risk and low-risk groups (Figure S6a,b). The results showed significantly higher infiltration of M0 macrophages (*p* < 0.01) in the high-risk group, while monocytes (*p* < 0.01) and CD8+ T cells (*p* < 0.05) exhibited significantly higher levels in the low-risk group. Spearman’s correlation analysis of infiltration levels versus the risk score revealed that monocytes, CD8+ T cells, activated B cells, and naive B cells were negatively correlated with the risk score, whereas M1 and M0 macrophages, activated dendritic cells, memory B cells, and eosinophils were positively correlated with the risk score (all *p* < 0.05) (Figure 5a–c).

**Figure 6:**
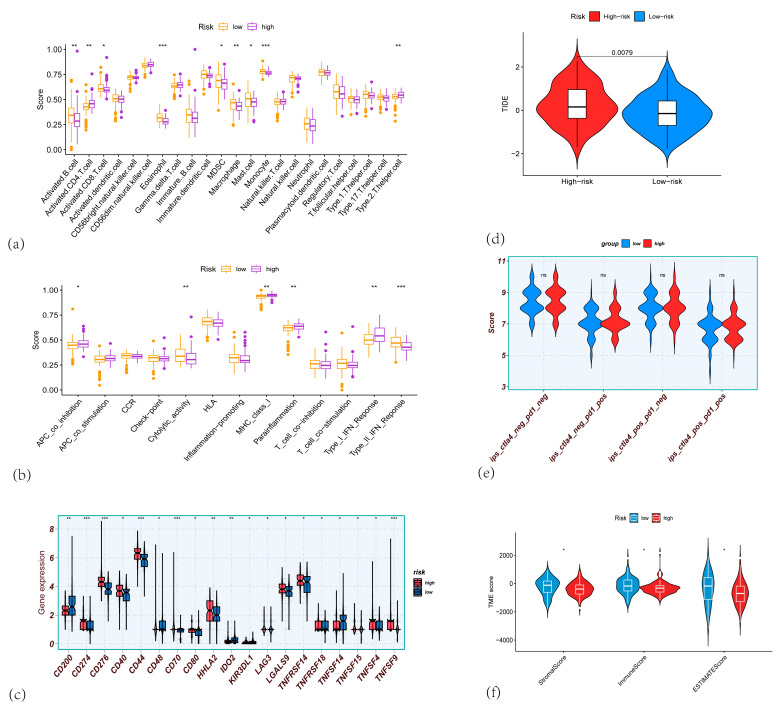
(**a**) Box plots showing the immune scores for various immune cells across high-risk and low-risk groups in PAAD. (**b**) Box plots showing the differences in immune-related function between the high-risk and low-risk groups. (**c**) Box plots showing the gene expression of immune checkpoint genes, comparing high-risk and low-risk PAAD patients. (**d**) Violin plots showing the TIDE scores between high-risk (red) and low-risk (blue) groups, indicating the potential of immune evasion in the two risk groups. (**e**) Violin plots comparing the IPS (Immune-related Pathway Signature) scores between high-risk and low-risk PAAD patients. (**f**) Violin plots showing the differences in ImmuneScore, StromalScore, and ESTIMATEScore, calculated by the ESTIMATE algorithm, comparing high-risk and low-risk PAAD patients. (* *p* < 0.05; ** *p* < 0.01; *** *p* < 0.001).

We further conducted single-sample Gene Set Enrichment Analysis (ssGSEA) to compare immune cell enrichment between the two risk groups. Compared to the high-risk group, the low-risk group showed significantly higher enrichment scores for eosinophils, monocytes, and macrophages (*p* < 0.001), and elevated but slightly less significant scores for activated B cells and macrophages (*p* < 0.01). The myeloid-derived suppressor cells (MDSCs) and mast cell scores were also significantly higher (*p* < 0.05) in the low-risk group. In contrast, the low-risk group displayed a notably lower abundance of activated CD4+ T cells (*p* < 0.001) and Type 2 T helper cells (*p* < 0.05) than the high-risk group (Figure 6a). As for immune functions, the low-risk group had significantly higher Type_II_IFN_Response and cytolytic activity scores, yet lower Type_I_IFN_Response, parainflammation, MHC_class_I, and APC_co_inhibition scores (Figure 6b). Regarding immune checkpoint expression, genes including CD274, CD276, CD44, and TNFSF9 were more highly expressed in the high-risk group (*p* < 0.001) (Figure 6c).

Using the ESTIMATE algorithm, we examined the relationship between the CARG signature and the tumor microenvironment in 177 PAAD patients from the TCGA (n = 177). ESTIMATE analysis indicated that stromal score (*p* = 0.027), immune score (*p* = 0.019), and ESTIMATE score (*p* = 0.02) were higher in the low-risk group (all *p* < 0.05). Immunotherapy, including immune checkpoint blockade (ICB) and cell-based treatments, has yielded durable clinical responses and transformative cancer therapies 999. However, there is currently no established immunotherapy regimen for pancreatic malignancies. Hence, unraveling the molecular mechanisms governing the immunosuppressive microenvironment is essential for improving immunotherapy outcomes and patient prognosis in PAAD. As TIDE offers a computational predictor of ICB responses, we applied the TIDE tool (http://tide.dfci.harvard.edu/) to assess the potential clinical efficacy of ICB for different risk scores (Figure 6f). According to TIDE, the group predicted to respond to immunotherapy had lower risk scores than the non-responding group (*p* = 0.00028), suggesting that high-risk patients are less likely to benefit from ICB (Figure 6d). Additionally, TCIA-based analysis revealed no statistically significant difference in immune scores between the high- and low-risk groups under various immune checkpoint inhibitor combinations (e.g., CTLA4 plus PD-1) (Figure 6e).

### 3.5. Functional Analysis of the CARG Gene Set

**Figure 7:**
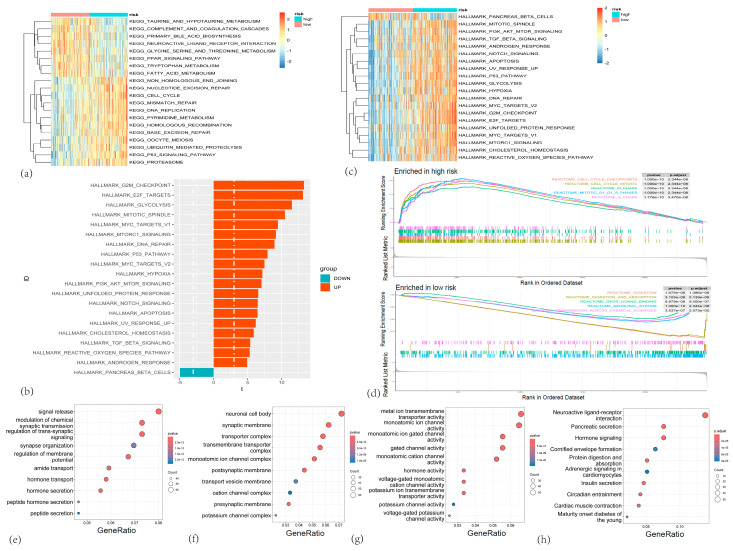
(**a**) Heatmap showing the enrichment of hallmark gene sets between high-risk and low-risk groups. (**b**) Bar plot depicting the number of differentially expressed hallmark gene sets between high-risk and low-risk groups. (**c**) Heatmap showing the enrichment of KEGG gene sets in high-risk and low-risk groups. (**d**) The important pathway of Gene set enrichment analysis (GSEA) plot comparing the high-risk and low-risk groups. (**e**–**g**) Bubble charts depicting Gene Ontology (GO) enrichment analyses of different genes in high-risk and low-risk groups. () Biological process (BP) terms; (**f**) cellular component (CC) terms; and (**g**) molecular function (MF) terms. (**h**) KEGG pathway enrichment analysis.

To investigate the biological behavior underlying different CARG risk scores, we performed functional annotation using the GSVA R package on TCGA-PAAD samples. Heatmap visualization of the GSVA-KEGG results (Figure 7c) showed significant enrichment of DNA repair pathways (e.g., non-homologous end joining, base excision repair, homologous recombination repair, nucleotide excision repair, mismatch repair), cell cycle and DNA replication pathways, the pentose phosphate pathway, the p53 signaling pathway, and proteasome pathway in the high-risk group. Conversely, pathways related to amino acid metabolism (e.g., valine, leucine, and isoleucine degradation; tryptophan metabolism; β-alanine metabolism), fatty acid metabolism, primary bile acid biosynthesis, taurine and hypotaurine metabolism, and the neuroactive ligand–receptor interaction pathway were significantly enriched in the low-risk group.

GSVA-HALLMARK analysis further revealed that cell cycle-related pathways, such as the mitotic spindle, DNA repair, mismatch repair, the unfolded protein response, G2M checkpoint, E2F targets, and MYC targets, were significantly enriched in the high-risk group, consistent with GSVA-KEGG findings and indicative of enhanced cell cycle progression. Additionally, stress and cell death-related pathways, including apoptosis and hypoxia, were also enriched. Notably, cancer-related pathways, such as PI3K/AKT/mTOR, Notch, p53, and TGF-β signaling, were more active in the high-risk group compared to the low-risk group (Figure 7a,b).

To further elucidate differences in gene expression associated with the CARG risk score, we identified differentially expressed genes (DEGs_2) between high-risk and low-risk samples and performed GSEA. GSEA showed that the high-risk group was primarily enriched in pathways related to the cell cycle, mitotic cell cycle, and keratinization (including the formation of the cornified envelope), whereas the low-risk group exhibited enrichment in pathways involved in synaptic transmission, G protein-coupled receptor activity, the nervous system, as well as digestion and absorption (Figure 7d).

Subsequently, we performed GO-KEGG enrichment analysis using the org.Hs.eg.db and clusterProfiler packages. The results indicated that, in terms of biological processes, genes were concentrated in signaling, secretion, and transport functions, such as signal release, regulation of synaptic transmission, and membrane potential. For cellular components, key structures included neuronal cell bodies, synaptic membranes, transporter complexes, transport vesicles, and ion channel complexes, potentially contributing to tumor microenvironment changes. As for molecular functions, the enriched terms were predominantly related to ion channels and transmembrane transport, including metal ion transmembrane transporter activity, gated channel activity, and single-ion gated channel activity. KEGG pathway analysis revealed that genes were mainly enriched in secretion, metabolic processes, and signaling pathways, including neuroactive ligand–receptor interaction, pancreatic secretion, and cAMP signaling (Figure 7e–h).

### 3.6. Mutation Analysis Result

**Figure 8:**
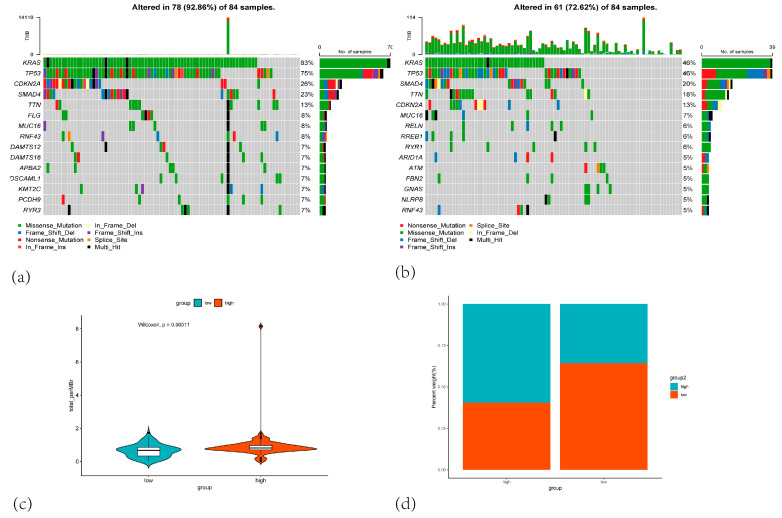
(**a**) Oncoprint of mutations in the top 10 most frequently altered genes in the high-risk group. (**b**) Oncoprint of mutations in the top 10 most frequently altered genes in the low-risk group. (**c**) Violin plot comparing the total number of mutations (total mutation count per megabase) between the low-risk (blue) and high-risk (orange) groups. (**d**) Stacked bar plot showing the percentage distribution of patients in the high-risk and low-risk groups based on mutation burden.

We further characterized the genomic mutation profiles associated with the CARG gene set, including mutation counts and mutation spectra, using the Maftools package in R to visualize mutation patterns in 177 PAAD patients from the TCGA-PAAD cohort. Notably, the key cell cycle regulator TP53 and the core signaling factor KRAS showed higher mutation rates in the high-risk group than in the low-risk group (TP53: 76% vs. 45%, KRAS: 83% vs. 46%, Figure 8a,b). Moreover, most KRAS mutations in the high-risk group were missense mutations that lead to constitutive activation of KRAS, which in turn activates MAPK/ERK and PI3K/AKT pathways, both of which are critical for cell proliferation, differentiation, and survival. This observation may partially explain the hyperactivated proliferative and differentiative signaling in the high-risk group. Additionally, we noted a higher frequency of CDKN2A mutations in the high-risk group and SMAD4 mutations in the low-risk group, suggesting potential distinct oncogenic processes. Regarding tumor mutation burden (TMB), the proportion of high-TMB samples was higher in the low-risk group than in the high-risk group, with a statistically significant difference (Figure 8c,d, *p* = 0.00011). This finding aligns with the hypothesis that high-risk patients may respond less favorably to immunotherapy.

### 3.7. Chemotherapy Response Prediction and the CARG-Based Risk Score

**Figure 9:**
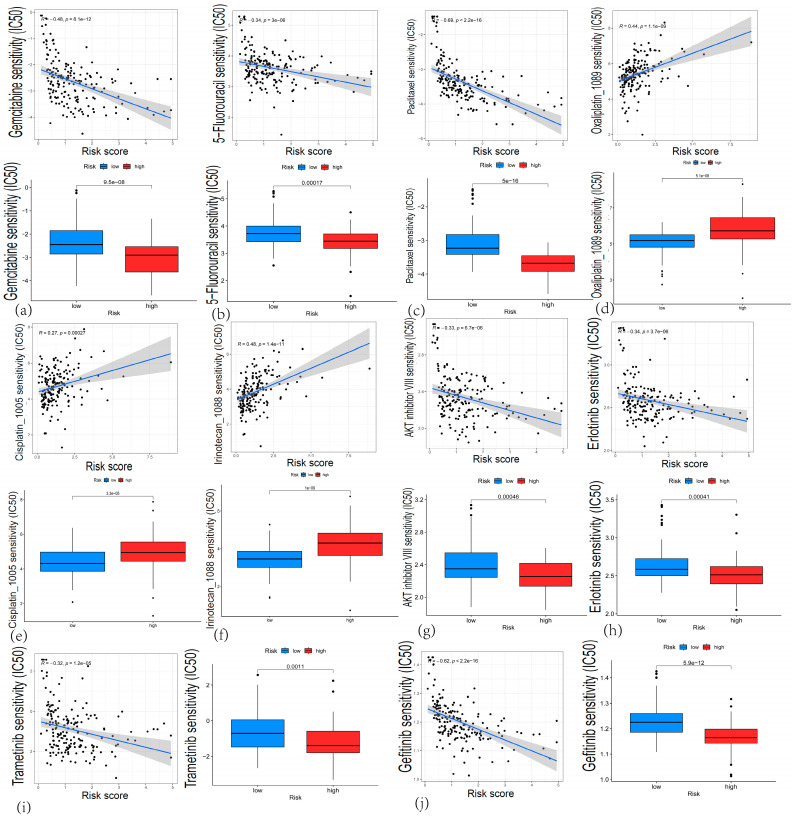
Drug sensitivity analysis stratified by high-risk and low-risk group. Correlation between risk score and IC50 values; IC50 distribution in high-/low-risk groups. (**a**) Gemcitabine; (**b**) 5-fluorouracil; (**c**) paclitaxel; (**d**) oxaliplatin; (**e**) cisplatin; (**f**) irinotecan; (**g**) AKT inhibitor VIII; (**h**) erlotinib; (**i**) trametinib; (**j**) gefitinib.

Using the Cancer Genome Project (CGP) cell line data, we predicted the IC50 values of commonly used chemotherapeutic agents for PAAD in different risk groups. Gemcitabine is a frontline therapy for pancreatic cancer [7]. Our analysis showed that its half-maximal inhibitory concentration (IC50) was significantly lower in the high-risk group compared to the low-risk group, suggesting increased sensitivity to gemcitabine-based chemotherapy among high-risk patients (Figure 9a). Similarly, 5-fluorouracil (5-FU), paclitaxel, AKT inhibitor VIII, gefitinib, trametinib (a MEK inhibitor), and erlotinib all exhibited lower IC50 values in the high-risk group (Figure 9b,c,g–j), indicating that these patients may also respond better to regimens containing these agents. Conversely, several key anti-pancreatic cancer drugs—namely irinotecan, oxaliplatin, and cisplatin—had markedly lower IC50 values in the low-risk group (Figure 9d–f), suggesting potential benefit for patients with low CARG risk scores.

### 3.8. Validation of the Five-Gene Signature Using Single-Cell Sequencing

**Figure 10:**
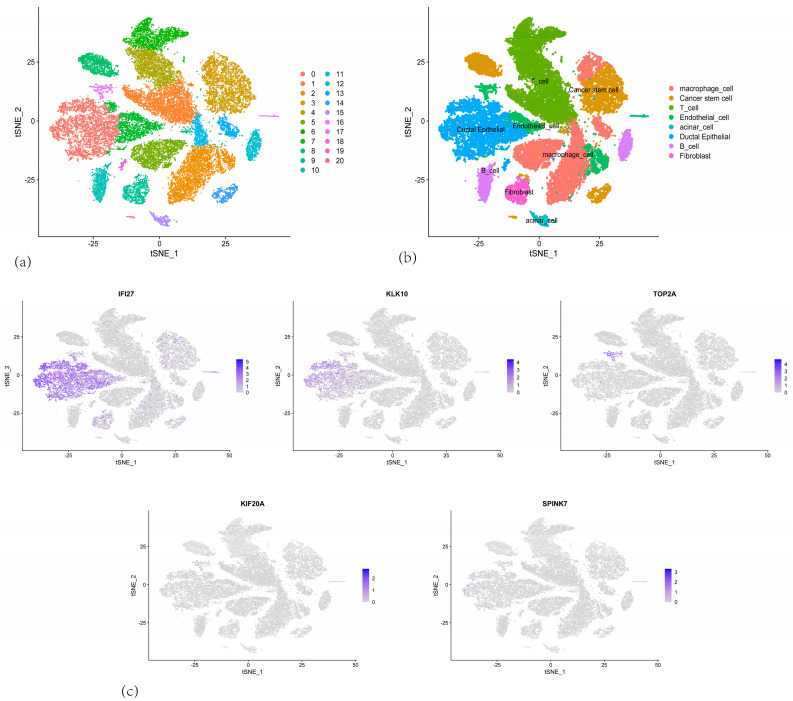
(**a**) t-SNE plot showing the clustering of single-cell RNA sequencing data from PAAD samples. (**b**) t-SNE plot with Cell type annotations. (**c**) t-SNE plot highlighting the expression of *IFI27*, *KLK10*, *TOP2A*, *SPINK7* and *KIF20A* in the single cells.

To further elucidate the role of the CARG signature in PAAD progression, we conducted single-cell RNA sequencing (scRNA-seq) analysis on GSE155698 to examine the expression profiles of CARGs within the malignant pancreatic tumor microenvironment. After quality control and filtering, 24,094 cells were obtained from 10 primary pancreatic tumors and grouped into 22 clusters (Figure 10a). Subsequent merging, clustering, and annotation classified these cells—based on known marker genes—into T cells, macrophages, endothelial cells, pancreatic ductal cells, acinar cells, endocrine cells, fibroblasts, B cells, and pancreatic tumor stem cells (Figure 10b).

We then visualized the expression patterns of the five centrosome amplification-related genes (*IFI27*, *KIF20A*, *KLK10*, *SPINK7*, and *TOP2A*) (Figure 10c). *IFI27* showed high expression in ductal cells and fibroblasts, whereas *KLK10* was strongly expressed in ductal cells, suggesting that *IFI27* and *KLK10* could be closely linked to the development of pancreatic ductal carcinoma. *TOP2A* was minimally expressed in B cells, and *SPINK7* and *KIF20A* had relatively low expression across tissue types.

### 3.9. Cell–Cell Communication Result

**Figure 11:**
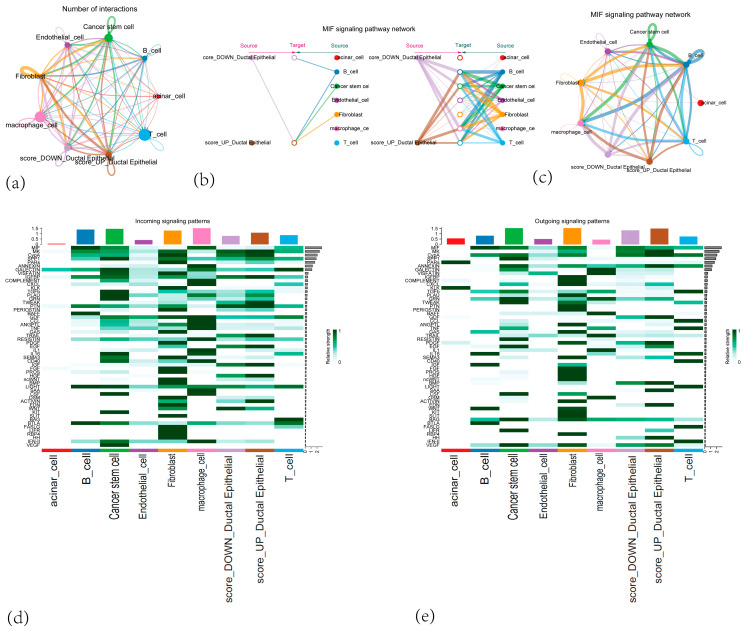
(**a**) A network graph showing the number of interactions between different cell types in the tumor microenvironment. (**b**) A hierarchical layout diagram of the MIF (Macrophage Migration Inhibitory Factor) signaling pathway network. (**c**) A network diagram of the MIF (Macrophage Migration Inhibitory Factor) signaling pathway network. (**d**) A heatmap displaying outgoing signaling patterns from the different cell types. (**e**) A heatmap displaying incoming signaling patterns to the different cell types.

To investigate intercellular interactions in the tumor microenvironment, we grouped the ductal epithelial cell subtype by high versus low module scores and analyzed the cellular communication network among various cell types in PAAD tumor tissues. Our findings demonstrated that high-scoring ductal epithelial cells, fibroblasts, and macrophages exhibited more active cross-talk, with complex intercellular connections among these cell subsets (Figure 11a). This observation points to a potential role for fibroblasts and macrophages in promoting malignant transformation.

We further examined the signaling pathways relayed from each cell subtype to others (Figure S7) as well as outgoing and incoming signals for each cell type (Figure 11d,e). The analysis indicated that each cell population possesses a complex network of incoming and outgoing signals. Notably, significant differences emerged in the MIF-(CD74 + CD44) and MIF-(CD74 + CXCR4) axes between high- and low-scoring ductal epithelial cell populations and B cells. Additionally, an MIF pathway network map was constructed, with each node representing a cell type and edge thickness indicating the intensity of MIF signal transmission (Figure 11b,c). This visualization underscores the intricate and vital role of MIF signaling in mediating cell–cell communication within the tumor microenvironment.

### 3.10. Spatial Transcriptomic Analyses of Five Core Genes

**Figure 12:**
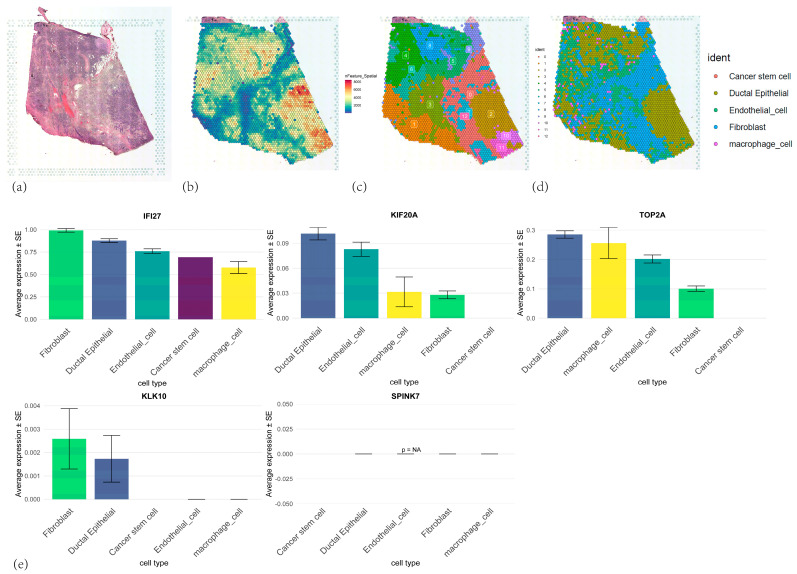
(**a**) Pancreatic tumor tissue section. (**b**) Spatial distribution patterns of UMIs and gene counts on the tissue section. (**c**) UMAP dimensionality reduction plot showing the clustering of ST from PAAD tissue. (**d**) Cell type annotation of the tissue section by RCTD deconvolution algorithm. (**e**) The expression levels of 5 core genes in different cell types.

**Figure 13:**
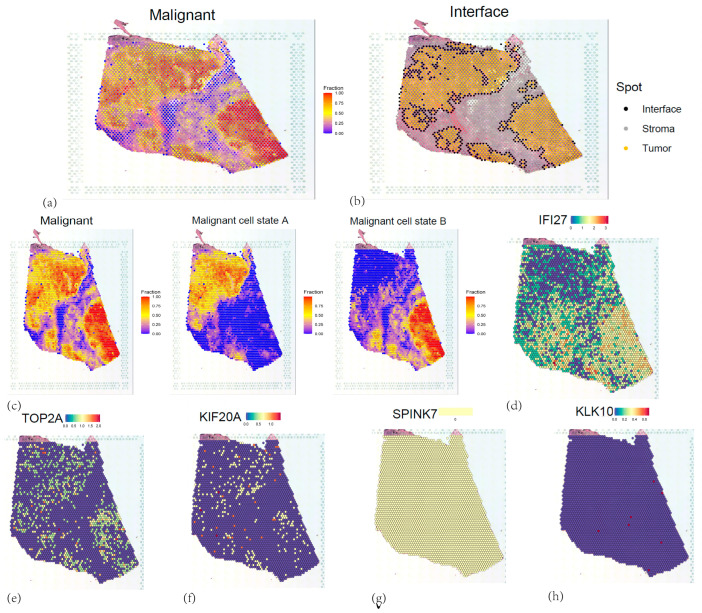
(**a**) Identification and distribution of malignant tumor cells. (**b**) Interface of malignant tumor cells. (**c**) Staging of malignant tumor cells. (**d**–**h**) Expression of 5 core genes.

**Figure 14:**
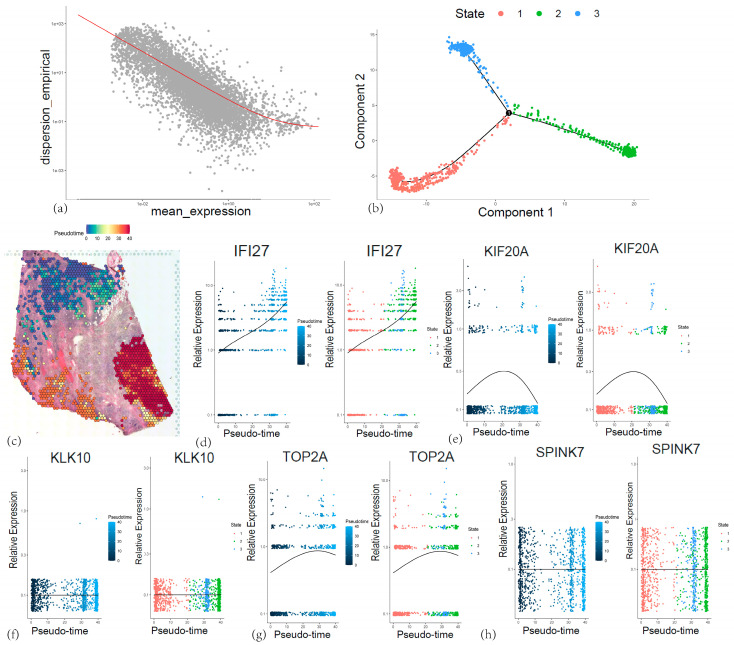
(**a**) Relationship between gene expression intensity and stability in ductal epithelial cells. (**b**) Branch states of ductal epithelial cell subpopulations. (**c**) Spatial distribution of pseudotime values, with color gradient reflecting the temporal progression of cellular differentiation. (**d**–**h**) Expression patterns of five core genes in relation to pseudotime.

The spatial distribution characteristics of 5 core genes in pancreatic cancer tissues were investigated through spatial transcriptomic analysis of ST data (GSE274557) (Figure 12a). RTCD deconvolution-based annotation was performed on the ST data using single-cell annotation data from GSE155698, resulting in annotated cell subtypes including cancer stem cells, ductal epithelial cells, endothelial cells, fibroblasts, and macrophages (Figure 12b–d). Spatial mapping revealed *IFI27*’s predominant localization in ductal epithelial regions and fibroblast regions, aligning with scRNA-seq profiles, while other genes exhibited dispersed low-expression patterns (Figure 12e and Figure 13d–h). SpaCET-based malignant subtyping (Figure 13a,b) identified *IFI27* as a hallmark of high-grade malignancy subcluster B (tumor purity > 80%, *p* < 1 × 10^−5^) (Figure 13c). Pseudotemporal trajectory analysis demonstrated Cell developmental trajectory (Figure 14a–c) and developmental-stage-specific dynamics. *IFI27* expression progressively escalated in late-stage malignant cells, *KIF20A/TOP2A* peaked during mid-pseudotime phases, whereas *KLK10/SPINK7* showed pseudotime-independent patterns, suggesting microenvironment-regulated expression (Figure 14d,e,g,f,h). Spatial colocalization networks revealed endothelia niches co-enriched with M2 macrophages and CAF, also, CAF co-enriched with M1 and M2 macrophages, corroborating their synergistic roles in tumor progression (Figure S8).

### 3.11. In PCR Validation of CARG-Related Prognostic Genes and HPA Immunohistochemistry in PAAD

**Figure 15:**
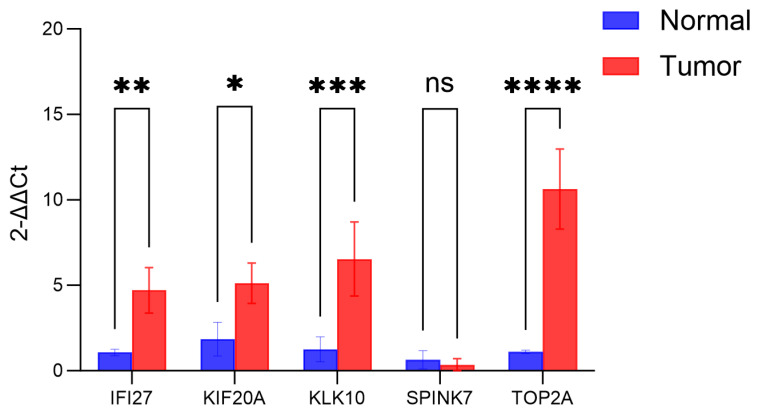
The expression levels of 5 core genes in pancreatic cancer tissues and normal tissues based on PCR analysis. (* *p* < 0.05; ** *p* < 0.01; *** *p* < 0.001; **** *p* < 0.0001).

To confirm the expression and function of the prognostic marker genes *IFI27*, *KIF20A*, *KLK10*, *SPINK7*, and *TOP2A*, we measured their mRNA levels by qRT-PCR. *IFI27*, *KIF20A*, *KLK10*, and *TOP2A* were highly expressed in tumor cells, supporting their potential as prognostic biomarkers in PAAD. Results showed that *IFI27*, *KIF20A*, *KLK10* and *TOP2A* were significantly upregulated in tumor cells relative to normal cells, whereas *SPINK7* showed no differential expression (Figure 15). The IHC results for proteins corresponding to the four genes (*IFI27*, *KIF20A*, *SPINK7*, and *TOP2A*) are available on the HPA. Based on IHC analysis, the distribution and staining intensity of these proteins in representative PAAD samples varied from low to high expression (Figure S9).

## 4. Discussion

We constructed a robust 5-gene survival prediction model based on centrosome amplification-related genes, achieving training set AUC values of 0.76–0.82 at 2, 3, 5 years and validation set AUC values of 0.74–0.82. Compared to published pancreatic cancer models—such as Song W’s anoikis-related model (training AUC 0.70–0.76, validation AUC 0.62–0.68 [8]), Deng J’s immunogenic autophagy model (training AUC 0.64–0.76, validation AUC 0.56–0.77 [9]), and Li Z’s immune-related model (training AUC 0.75–0.79, validation AUC 0.59–0.83 [10])—our 5-gene signature demonstrates significantly superior predictive robustness in both training (AUC = 0.76–0.82) and independent validation cohorts (AUC = 0.74–0.82), outperforming existing models (e.g., anoikis/autophagy-related). Therefore, this CARG-based signature may serve as a valuable biomarker for PAAD diagnosis and prognosis. This study pioneers the first centrosome amplification (CE)-driven survival prediction model for pancreatic cancer, integrating multi-omics evidence (transcriptomics + single-cell + spatialomics) to reveal CE genes promote malignant progression by driving genomic instability and remodeling the tumor microenvironment.

Our CARG-based signature comprises *IFI27*, *KIF20A*, *KLK10*, *SPINK7*, and *TOP2A*. *IFI27* (Interferon Alpha Inducible Protein 27) has been proposed as a potential prognostic biomarker in pancreatic cancer, where its overexpression correlates with increased cell proliferation, metastasis, and malignant progression [11]. Literature indicates that functional annotations of *IFI27* in pancreatic cancer reveal its close association with cellular immunity and metabolism, particularly glycolysis, and increased *IFI27* expression is associated with reduced CD8+ T cells and increased M2-type macrophages. *IFI27* is transcribed as a type I interferon (IFN-I) stimulated gene (ISG) mediated by the STAT1/STAT2/IRF9 complex, and activation of the interferon-alpha receptor and increased mRNA levels of ligands and receptors in the TGFB pathway play important roles in the PDAC tumor microenvironment [12].

*KIF20A* (kinesin family member 20A) binds to microtubules and generates mechanical force through ATP hydrolysis, playing a crucial role in cell division [13]. Overexpression of *KIF20A* in pancreatic tumors typically correlates with increased invasiveness and poor prognosis [14,15], suggesting its potential as a therapeutic target.

*KLK10* (kallikrein-related peptidase 10), also known as normal epithelial cell–specific 1 (NES1). Elevated *KLK10* expression is associated with poor prognosis in pancreatic cancer. *KLK10* contributes to the pancreatic tumor microenvironment by modulating cellular motility [16]. Furthermore, *KLK10* enhances epithelial–mesenchymal transition (EMT) and activates FAK-SRC-ERK signaling, thereby promoting invasion and metastasis in pancreatic cancer, further implicating *KLK10* in PDAC initiation and progression [17].

Our data indicate that *SPINK7* is highly expressed in pancreatic cancer tissues and correlates with poor prognosis. However, few studies have addressed *SPINK7* in PAAD, and no significant relationship has been reported between *SPINK7* short tandem repeat (STR) polymorphisms and reduced pancreatic cancer risk or improved overall survival [18].

*TOP2A* (topoisomerase IIα) is overexpressed in pancreatic cancer and linked to worse survival outcomes [19]. By activating the Wnt/β-catenin pathway, *TOP2A* accelerates pancreatic tumor proliferation and migration [20].

Centrosome amplification is tightly controlled throughout the cell cycle. However, when cell division fails, disrupted CDK activity triggers centrosome expansion, resulting in multipolar spindle formation and erroneous chromosome segregation—ultimately leading to aneuploidy and malignant transformation [21]. Consistent with these findings, our study showed that patients in the high-CARGs-risk group exhibited significant enrichment in cell cycle-related pathways, DNA repair, and PI3K/AKT/mTOR signaling, highlighting a close association between cell cycle dysregulation and centrosome amplification. In addition, high-risk patients demonstrated greater resistance to DNA synthesis inhibitors (gemcitabine, 5-FU), platinum-based drugs (oxaliplatin and cisplatin; these form platinum–DNA adducts that block DNA replication and transcription), and the topoisomerase I inhibitor irinotecan [22,23,24,25]. Enhanced DNA repair may facilitate the correction of irinotecan-induced DNA breaks, thereby diminishing its cytotoxic impact [26], it correlates with the enrichment of DNA repair pathways in the high-risk group. Furthermore, patients with high CARGs scores showed elevated activity in the PI3K/AKT/mTOR, Notch, p53, and TGF-β pathways. This suggests that certain tyrosine kinase inhibitors (TKIs)—such as gefitinib and erlotinib, which target the EGFR pathway [27]—may exert enhanced antitumor effects in the high-risk group.

A high KRAS mutation frequency is one of the most prominent characteristics of pancreatic cancer. The MAPK pathway, which is closely integrated with the cell cycle, orchestrates Ras activation to promote tumor cell proliferation, survival, and migration [28]. The Ras oncogene can signal centrosome amplification through cyclin D1/Cdk4 and Nek2, promoting tumorigenesis [29]. TP53, a tumor suppressor gene closely linked to cell cycle control, has been implicated in early centrosome amplification, with some studies suggesting that its function is lost due to both the loss of wild-type TP53 expression and hotspot mutations [30]. In our study, KRAS and TP53 mutations were significantly more frequent in the high-risk group than in the low-risk group, further affirming that TP53 and KRAS mutations play critical roles in centrosome amplification.

Our study found that the high-risk group exhibited elevated infiltration of M0 macrophages, and several immune checkpoints, including CD274 (PD-L1), CD276, CD44, and TNFSF9, were also elevated in patients with high CARGs scores, which may be associated with the immunosuppressive microenvironment characteristics of pancreatic cancer. Studies have shown that tumor-associated macrophages can induce the expression of pro-inflammatory cytokines and upregulation of PD-L1 in M0 macrophages through IL-6/STAT3 and TLR4 signaling pathways, exerting an immunosuppressive phenotype related to programmed cell death ligand 1 (PD-L1) expression, thereby contributing to the formation of an immunosuppressive microenvironment, which drive ICB resistance [31]. Meanwhile, the significant depletion of CD8+ T cells in the high-risk group may correspond to the prevalent “immune desert” phenotype in PAAD, where desmoplastic extracellular matrix (ECM) and immunosuppressive cells physically block the infiltration of cytotoxic T cells [32]. Previous research indicates that centrosome amplification can induce spindle multipolarity during cell division, leading to chromosomal missegregation and ultimately chromosomal instability (CIN) [33]. CIN tumors show activation of suppressive inflammatory pathways (including cGAS/STING/APP), inducing immunosuppressive signals such as TGF-β and IL-10 that exhibit defective MHC class I antigen presentation [34]. Also, our findings demonstrated that, from an immune-function perspective, the low-risk group exhibited significantly higher Type II IFN Response and cytolytic activity than the high-risk group, whereas the Type I IFN Response, para-inflammation, MHC class I expression, and APC co-inhibition scores were significantly lower than those of the high-risk group. These observations imply that the low-risk group has stronger antitumor immunity, while the high-risk group displays more pronounced immunosuppressive features. Predictions of ICB therapy indicate that high-risk tumors may experience greater immune escape, leading to inferior responses to ICB. Thus, the CARGs score could serve as a biomarker to identify patients more likely to benefit from such treatments.

Our single-cell analysis revealed that CARGs-related genes are primarily expressed in ductal epithelial cells, with *IFI27* and *KLK10* exhibiting high expression in pancreatic ductal cells, and *IFI27* also presenting high expression in fibroblasts. These findings suggest a strong association of these genes with malignant transformation and progression of ductal epithelial cells in pancreatic cancer. By contrast, these genes showed relatively low expression in immune cells (T cells, B cells, macrophages) and cancer stem cells, possibly reflecting the immunologically “cold tumor” property of pancreatic cancer [7], where immune cell activity is suppressed by a strong immunosuppressive microenvironment. Cell–cell communication analysis highlighted complex interactions between cell populations, particularly elevated crosstalk among high-scoring ductal epithelial cells, fibroblasts, and macrophages. Notably, the MIF signaling pathway was significantly implicated, suggesting a protumor function for fibroblasts—especially cancer-associated fibroblasts (CAFs)—and macrophages, as well as a pivotal role for MIF in modulating immune responses and promoting pancreatic tumor cell proliferation.

The pancreatic tumor microenvironment (TME), constituting the bulk of tumor volume, encompasses fibroblasts, extracellular matrix (ECM) components, immune cells, nerves, and endothelial cells that collectively shape tumor cell phenotypes. Its complexity arises from heterogeneous cell–cell interactions and spatial heterogeneity features [35]. Spatial transcriptomics revealed *IFI27*’s predominant localization in ductal epithelial regions (aligning with scRNA-seq data), while *KLK10/SPINK7* exhibited microenvironment-regulated expression patterns unrelated to pseudotime. SpaCET analysis identified *IFI27* as a hallmark of high-grade malignancy subcluster B, with its expression escalating in late-stage malignant cells. *IFI27* is highly expressed in cell-rich regions, and carcinoma-associated fibroblasts (CAFs) isolated from these cell-infiltrated subregions display enhanced motility and greater tumor-proliferative capacity [36]. The tumor-suppressive potential of immune cells originates from crosstalk with other TME populations—such as macrophages in NKT cell contexts and fibroblasts in Treg contexts—demonstrating functionally significant heterogeneous interactions that drive distinct tumor evolutionary outcomes [37]. Spatial colocalization networks confirmed co-enrichment of endothelial niches with M2 macrophages and CAFs, as well as CAF-M1/M2 macrophage co-enrichment, corroborating their synergistic roles in tumor progression. Critically, pancreatic tumor-associated macrophages (TAMs) utilize mitochondrial bioenergetic programming fueled by fatty acid metabolism [38], further emphasizing metabolic–immune crosstalk within spatially organized niches.

## 5. Limitations

Firstly, this research primarily focused on *database bioinformatics analysis* for gene expression validation, lacking *functional validation of genes*. Secondly, due to *sample size limitations* in PCR experiments, validation of partial gene expression failed to achieve robust results; future studies require expanded cohorts for verification. Although the model’s efficacy was confirmed using GEO datasets, the absence of an *independent external validation cohort* compromises assessment of its generalizability. We plan to establish *center-based biobanks* to construct a powerful external validation cohort. Finally, *computer-predicted drug sensitivity profiles* lack in vitro *cellular or* ex vivo *organ validation*. Subsequent work will leverage biobanks to experimentally verify these computational predictions.

## 6. Conclusions

Overall, our study offers the first systematic analysis of centrosome amplification in PAAD that shows significant diagnostic value in diagnosing and prognosticating PAAD patients. Collectively, a comprehensive evaluation of centrosome amplification-associated genes in PAAD advances our understanding of the disease’s mechanisms and paves the way for innovative therapeutic interventions.

## Data Availability

Original contributions presented in the study are included in public repository. Further inquiries can be directed to the corresponding author.

## Supplementary Materials

The following supporting information can be downloaded at: https://www.mdpi.com/article/10.3390/cancers17182983/s1, Figure S1. PCA plot of TCGA-PAAD patients based on the 23 CARGs signature in tumor and normal tissue in 2D and 3D; Figure S2. Bubble charts depicting Gene Ontology (GO) enrichment analyses of the 23 overlapping genes. (a) Biological process (BP) terms; (b) Cellular component (CC) terms; and (c) Molecular function (MF) terms. (d) KEGG pathway enrichment analysis; Figure S3. Forest plot from multivariate Cox proportional hazards analysis of 5 genes in PAAD; Figure S4. (a) Univariate Cox proportional hazards regression analysis of clinical features and risk score in the TCGA-PAAD cohort. (b) multivariate Cox proportional hazards regression analysis of clinical features and risk score in the TCGA-PAAD cohort; Figure S5. (a–e) Boxplot showing the 5 genes expression distribution based on T,N,tumor stage. Figure S6. (a) Stacked bar plot showing the proportion of different immune cell types across the PAAD patients in TCGA. (b) Violin plots depicting the distribution of immune cell fractions across high-risk (red) and low-risk (blue) groups. Figure S7. A heatmap showing the interaction probabilities between different signaling pathways and cell to cell relationship. Figure S8. The spatial colocalization characteristics and correlations of different cell types in the tumor microenvironment. Figure S9. Protein expression levels of IFI27, KIF20A, SPINK7 and TOP2A in PAAD tumor tissues.
