# Supplementary material for: Development and Validation of a Centrosome Amplification-Related Prognostic Model in Pancreatic Cancer: Multi-Omics Guided Risk Stratification and Tumor Microenvironment
Source: Cancers (Basel). 2025 Sep 12;17(18):2983. doi: 10.3390/cancers17182983 (PMC12468691; doi:10.3390/cancers17182983)
Supplement: Supplementary file 1 [file cancers-17-02983-s001.zip › cancers-3831466-supplementary.pdf]

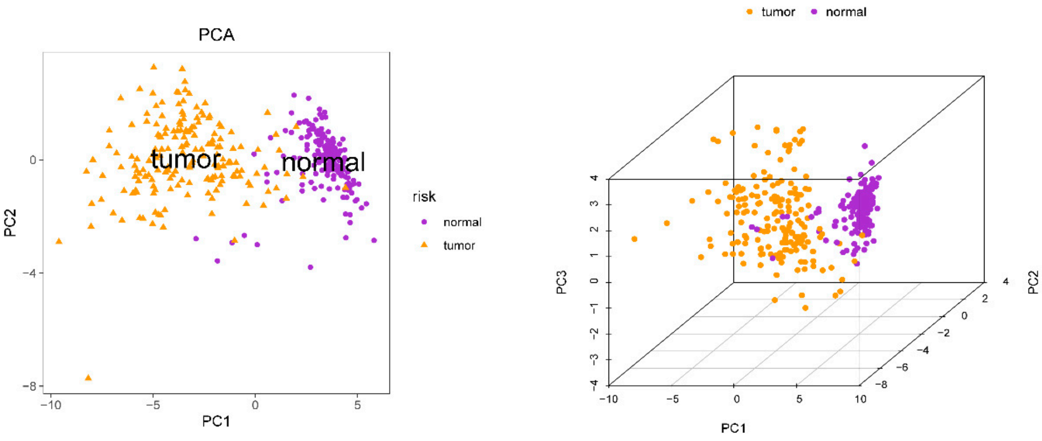

Figure S1. PCA plot of TCGA-PAAD patients based on the 23 CARGs signature in tumor and normal tissue in 2D and 3D.

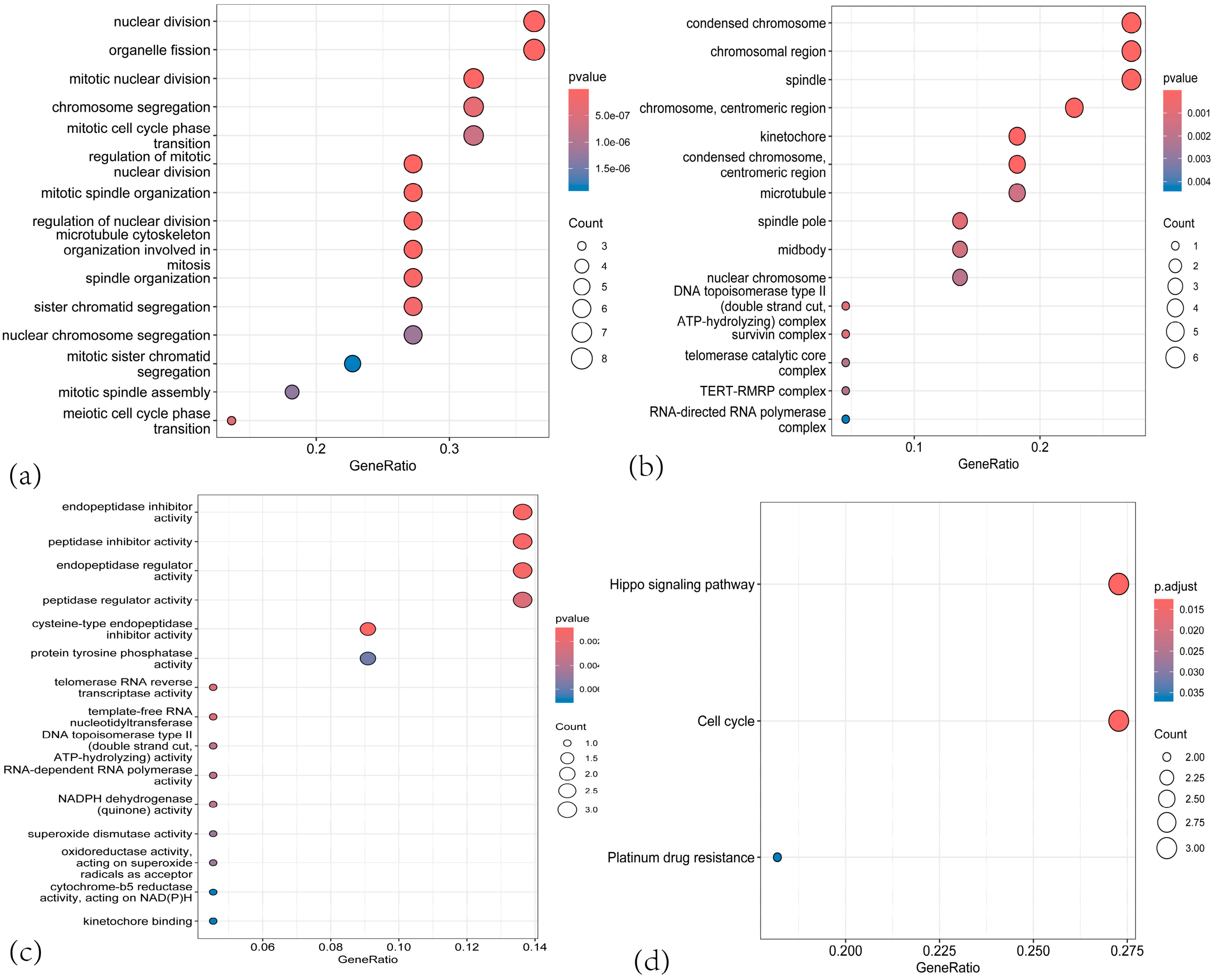

Figure S2. Bubble charts depicting Gene Ontology (GO) enrichment analyses of the 23 overlapping genes. (a) Biological process (BP) terms; (b) Cellular component (CC) terms; and (c) Molecular function (MF) terms. **(d)** KEGG pathway enrichment analysis.

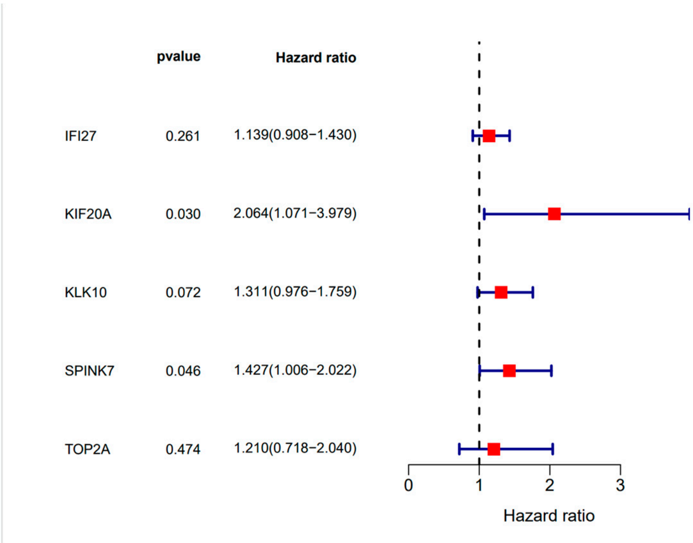

Figure S3. Forest plot from multivariate Cox proportional hazards analysis of 5 genes in PAAD.

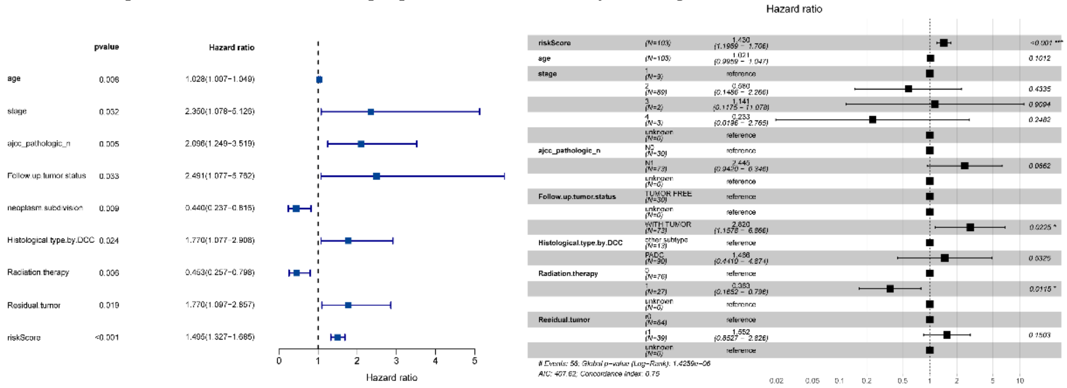

(a) (b)

Figure S4. (a) Univariate Cox proportional hazards regression analysis of clinical features and risk score in the TCGA-PAAD cohort.(b) multivariate Cox proportional hazards regression analysis of clinical features and risk score in the TCGA-PAAD cohort.

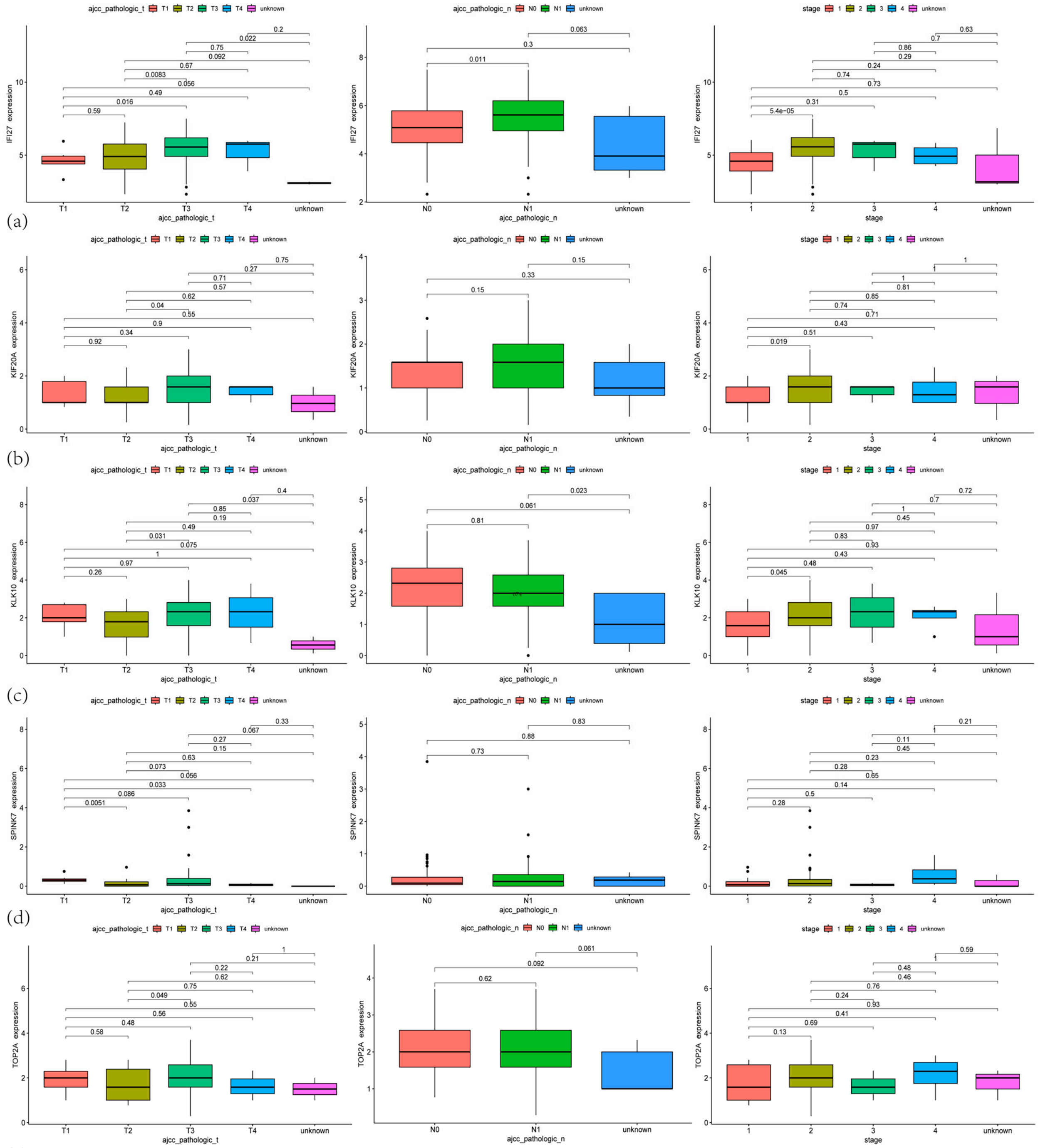

Figure S5.(a-e) Boxplot showing the 5 genes expression distribution based on T,N,tumor stage.

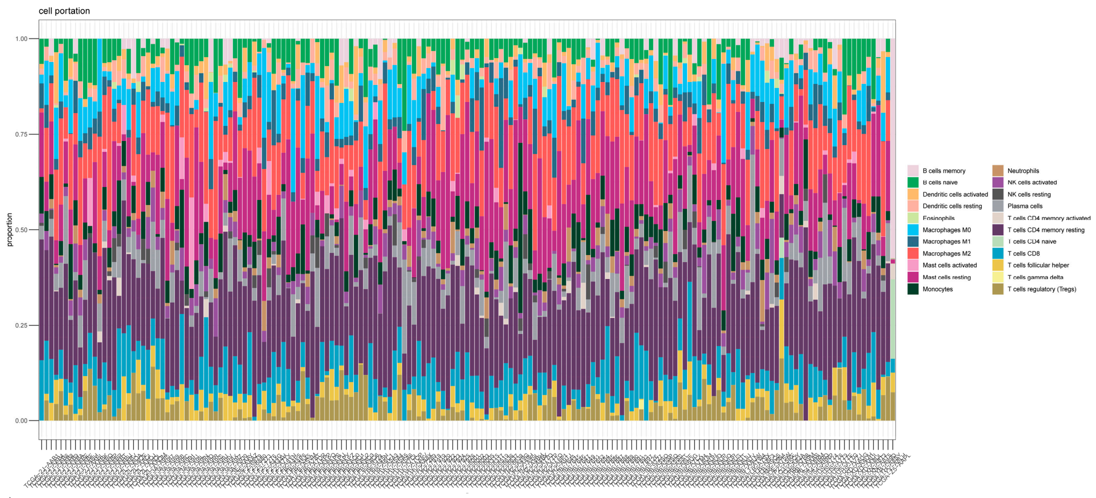

(a)

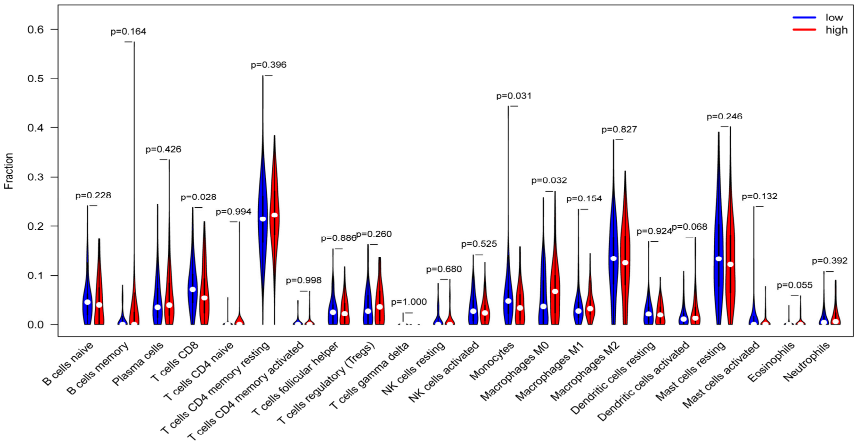

(b)

Figure S6. (a) Stacked bar plot showing the proportion of different immune cell types across the PAAD patients in TCGA. (b) Violin plots depicting the distribution of immune cell fractions across high-risk (red) and low-risk (blue) groups.

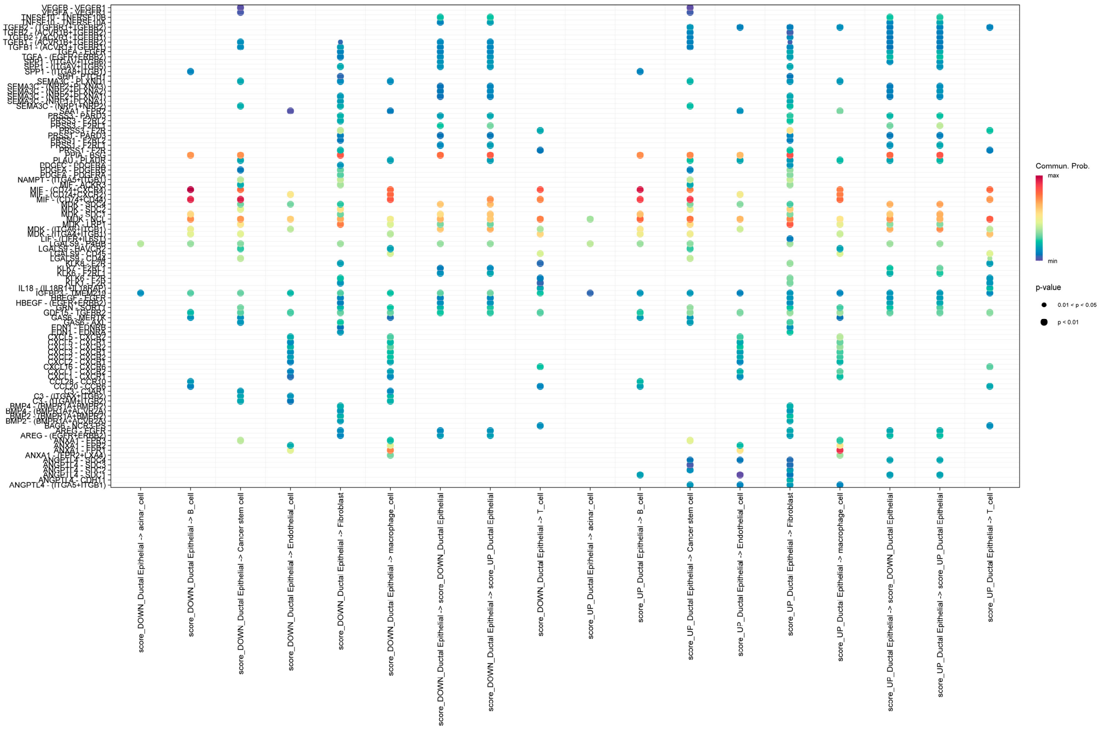

Figure S7. A heatmap showing the interaction probabilities between different signaling pathways and cell to cell relationship.

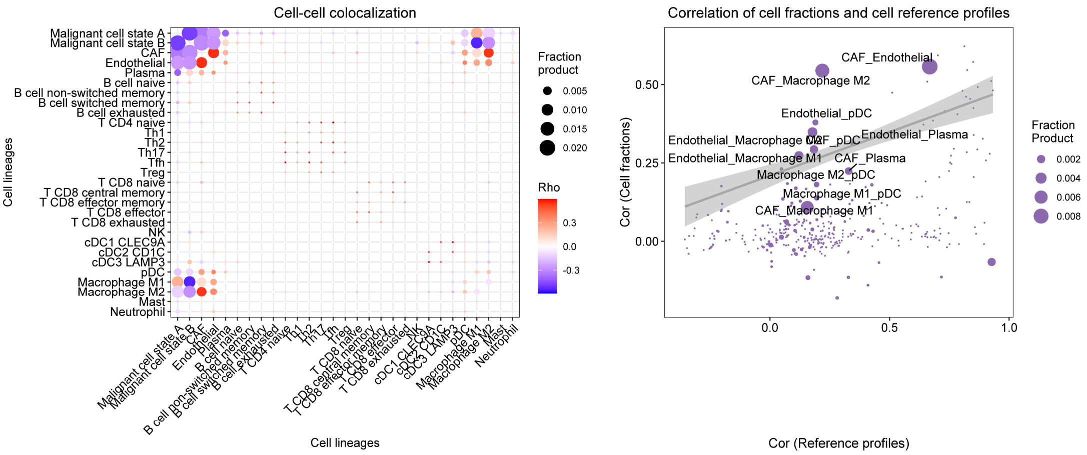

Figure S8. The spatial colocalization characteristics and correlations of different cell types in the tumor microenvironment.

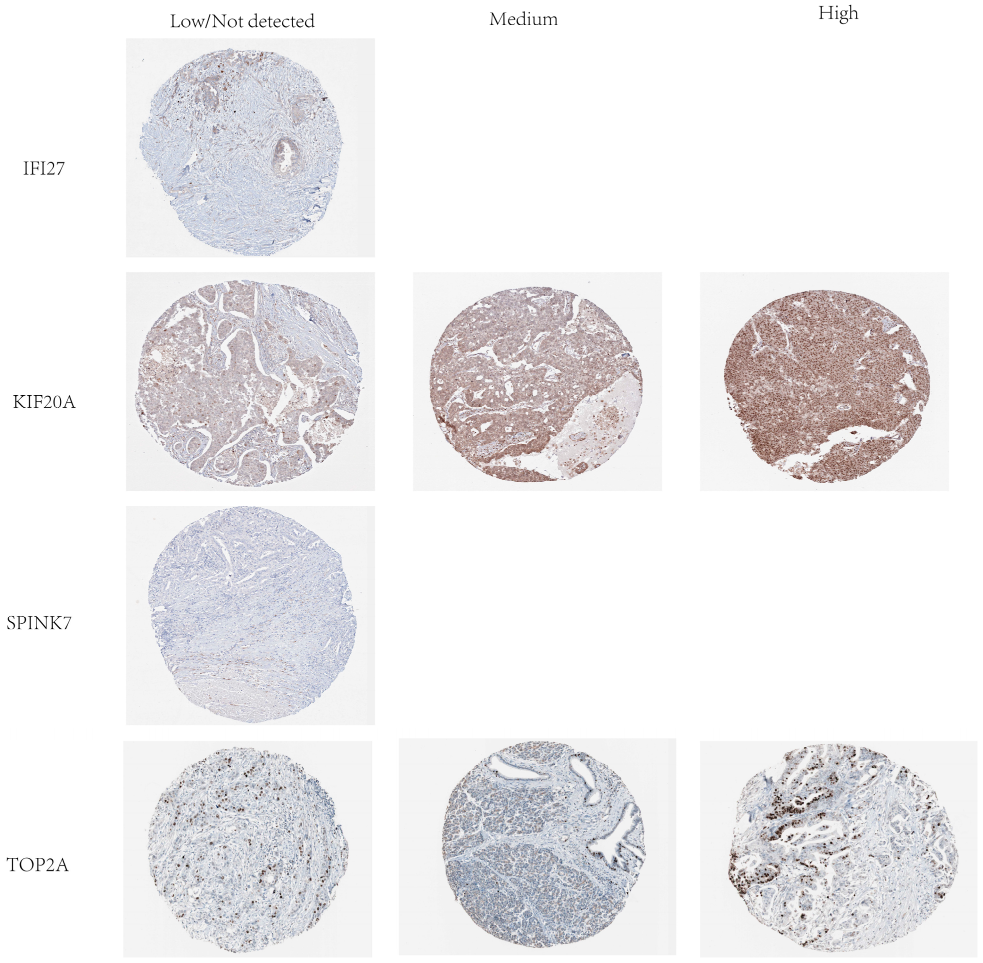

Figure S9. Protein expression levels of IFI27, KIF20A, SPINK7 and TOP2A in PAAD tumor tissues.
